# Efficient and Selective Biosynthesis of a Precursor-Directed FK506 Analogue: Paving the Way for Click Chemistry

**DOI:** 10.1021/acs.jnatprod.4c00394

**Published:** 2025-03-10

**Authors:** Dušan Goranovič, Branko Jenko, Barbara Ramšak, Ajda Podgoršek Berke, Leon Bedrač, Jaka Horvat, Martin Šala, Damjan Makuc, Guilhermina M. Carriche, Luana Silva, Aleksandra Lopez Krol, Alen Pšeničnik, María Beatriz Durán Alonso, Martina Avbelj, Stojan Stavber, Janez Plavec, Tim Sparwasser, Rolf Müller, Gregor Kosec, Štefan Fujs, Hrvoje Petković

**Affiliations:** # Acies Bio, d.o.o., 1000 Ljubljana, Slovenia; & University of Ljubljana, Biotechnical Faculty, Department of Food Science and Technology, 1000 Ljubljana, Slovenia; ¥ National Institute of Chemistry, 1000 Ljubljana, Slovenia; † Institute of Medical Microbiology and Hygiene and Research Center for Immunotherapy (FZI), University Medical Center of the Johannes Gutenberg-University, Mainz 55131, Germany; 1 Institute of Infection Immunology, TWINCORE, Centre for Experimental and Clinical Infection Research, a Joint Venture Between the Medical School Hannover (MHH) and the Helmholtz Centre for Infection Research (HZI), Hannover 30625, Germany; Ω Department of Biochemistry and Molecular Biology and Physiology, University of Valladolid, 47005 Valladolid, Spain; ∑ Department of Physical and Organic Chemistry, Jožef Stefan Institute, 1000 Ljubljana, Slovenia; ⊥ EN → FIST Centre of Excellence, Trg Osvobodilne fronte 13, 1000 Ljubljana, Slovenia; □ Faculty of Chemistry and Chemical Technology, University of Ljubljana, Večna pot 113, 1000 Ljubljana, Slovenia; 2 Helmholtz Institute for Pharmaceutical Research Saarland (HIPS), Helmholtz Centre for Infection Research (HZI), and Department of Pharmacy, Saarland University, 66123, Saarbrücken, Germany; § Centre of Excellence for Integrated Approaches in Chemistry and Biology of Proteins (CIPKeBiP), Jamova 39, SI-1000 Ljubljana, Slovenia

## Abstract

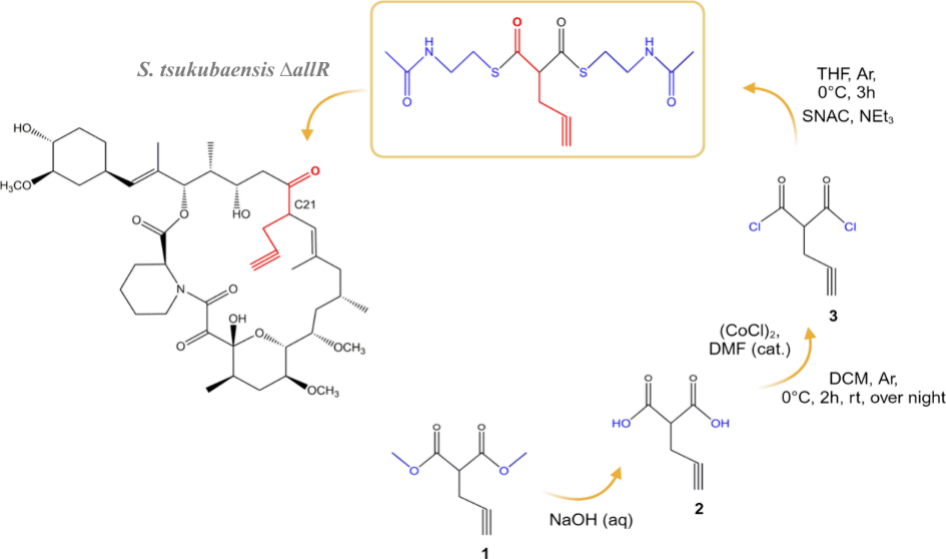

The medically important immunosuppressant FK506 is a structurally complex macrolactone biosynthesized by a combined polyketide synthase and a nonribosomal peptide synthetase enzyme complex. Its acyltransferase domain 4 (AT4) selects an unusual extender unit, resulting in an allyl moiety on carbon 21 of the macrolactone backbone. Based on the AT4 domain, chemobiosynthetic processes have been developed that enable the introduction of diverse moieties at the carbon 21 position. However, the novel moieties that were introduced into the polyketide backbone are chemically inert. Reported here is a novel and efficient chemobiosynthetic approach that ensures high titer of an FK506 analogue containing a propargyl moiety. The novel FK506 analogue displays lower immunosuppression activity than FK506 with significantly reduced cytotoxicity. More importantly, the propargyl moiety contains a terminal alkyl group, which makes click chemistry reactions possible; this approach may potentially be translated to other medically important drugs of polyketide origin.

Polyketides (PKs) are a large group of biogenetically related compounds with a diverse spectrum of activities; they are biosynthesized by the large group of closely related polyketide synthase (PKS) enzymes that display an enormous structural diversity.^1^ A particularly important PK group is constituted by structurally closely related macrolactones, such as rapamycin and the FKBP12-binding compounds FK506 and FK520. These metabolites exhibit a broad spectrum of pharmacological activities including immunomodulation, anticancer, and neuroprotection properties.^2^ Due to its powerful and selective immunosuppression activity, FK506 has been widely used to prevent organ rejection^3,4^ and, to some extent, in the treatment of inflammation-related conditions^5^ such as atopic dermatitis.^6^

Despite their potent activity, PKs frequently require further optimization by structural modification to improve their pharmacokinetic and pharmacodynamic properties. This is most often carried out by semisynthetic approaches; on the other hand, these are often very difficult to carry out, due to the structural complexity of natural products.

Precursor-directed chemobiosynthesis remains a valuable and industrially important method for obtaining new analogues of promising therapeutic molecules.^7^ Chemobiosynthesis is carried out by feeding a synthetic precursor to an engineered strain that has been deprived of one of its naturally occurring intermediates or that is unable to synthesize a building block needed for the biosynthesis of the target metabolite. Instead of the natural building block, synthetic precursors with different structures are fed into the culture of an engineered strain during the biosynthesis, thus resulting in the formation of a structural analogue of the target natural product.^7^ This approach has been used, for example, for the large-scale production of the antiparasitic PK doramectin, where unnatural cyclohexane carboxylic acid is incorporated instead of the natural starter unit.^8^ A number of unnatural precursor-derived chemobiosynthesis processes have also been developed for the biosynthesis of different PKs, including FK506 and rapamycin.^9−12^

FK506 (Tacrolimus, Figure 1) is a macrocyclic polyketide produced by a hybrid type I polyketide synthase (PKS)/nonribosomal peptide synthetase (NRPS) system of *Streptomyces tsukubaensis* and some other *Streptomyces* species.^12^ Typically, the PKS complex type I is composed of modules that contain domains with different activities, including acyltransferase (AT), ketosynthase (KS) and acyl-carrier protein (ACP), which are all obligatory domains present in every functional extender module.^13^ In the PKS type I complex, such as that found in the FK506 biosynthetic gene cluster (BGC) in *Streptomyces tsukubaensis*, acyltransferase (AT) domains are responsible for the selection and incorporation of simple monomeric building blocks. Extender AT domains usually exhibit a strict specificity toward a single α-carboxyacyl-CoA building block. Interestingly, however, AT domains associated with crotonyl-CoA carboxylase/reductase (CCR)-generated extender units can show relaxed specificity, and thus frequently give rise to PK structure diversification.^14^ This is also the case for the AT4 domain of the FK506 PKS, which most often incorporates the unusual extender unit allylmalonyl-CoA (Figure 1).^15,16^

**Figure 1:**
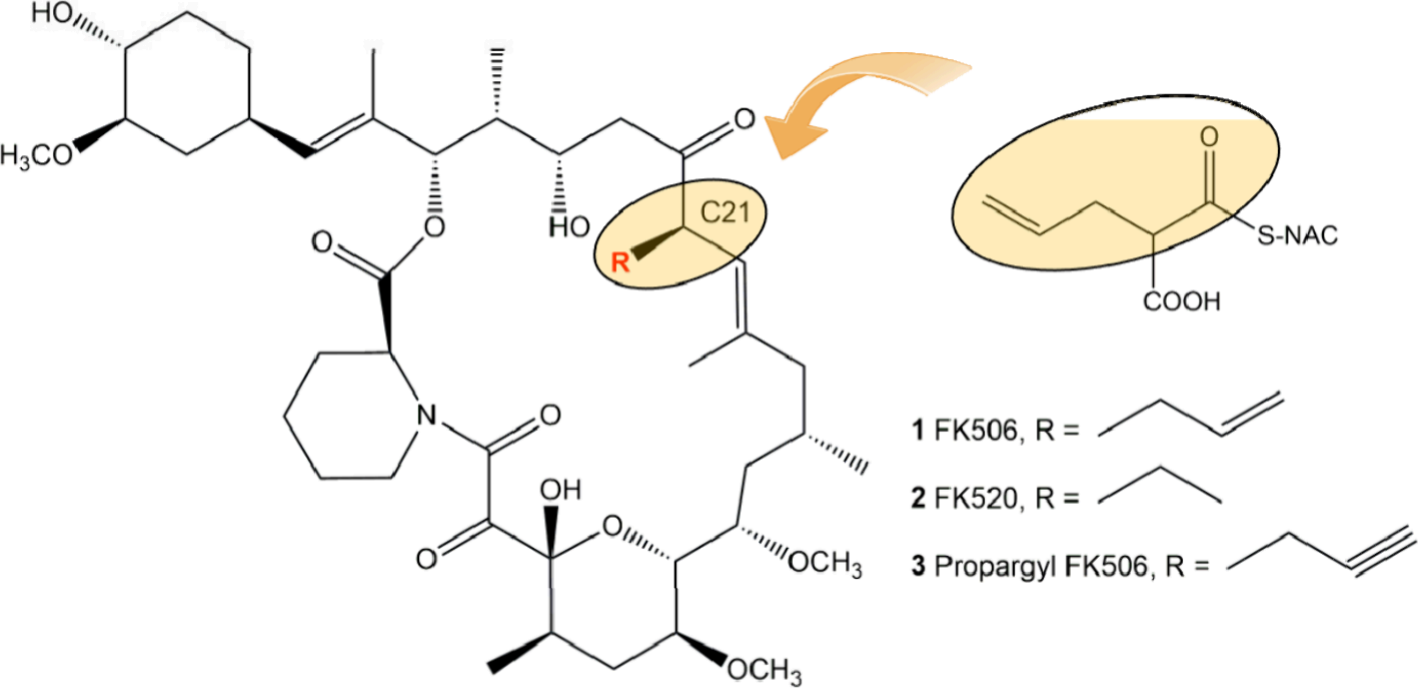
Structure of FK506 and the propargyl-FK506 analogue, showing the atom numbering used in our study.

The selective introduction of novel extender units would bring a much larger degree of PK diversification, and thus, the relaxed specificity of the acyltransferase (AT) domain in a typical PKS module would significantly increase the potential for novel chemobiosynthetic processes. With a few exceptions, AT domains remain stringent catalytic selectivity, predominantly favoring malonyl-CoA and methylmalonyl-CoA, while exhibiting lower preference for substrates such as ethylmalonyl-CoA and methoxymalonyl-CoA. Rarely do AT domains display selectivity for unusual extender units, including FK506 (allylmalonyl-CoA^16^) and stambomycin (hexanoyl-CoA^17^).

A feature of the AT4 domain from FkbB of FK506 PKS is its selectivity to accept unusual allylmalonyl-CoA, propylmalonyl-CoA, and ethylmalonyl-CoA extender units (Figure 1), but not usual methylmalonyl-CoA and malonyl-CoA extender units. This is the reason why the AT4 domain from the FK506 PKS/NRPS complex, which shows relaxed specificity for unusual acyl-CoA extender units, represents a very interesting model system that has been the focus of a number of studies in the past.^15,16^

Following the identification of the origin of the unusual extender unit allylmalonyl-CoA at position C21 of FK506,^16^ chemobiosynthesis procedures^15,18^ were developed by applying N-acetylcysteamine ester (SNAC) precursors, which are accepted by AT modules due to their similarity with native coenzyme-A-activated extender units.^19,20^ However, most of the unnatural extender units (precursors) incorporated to position C21 of FK506 have resulted in the introduction of structural moieties which are very difficult to chemically modify. In addition, the efficacy of these chemobiosynthesis processes is often very low, hence generating very low titers of the target products (often, in nanomolar quantities), which make this kind of bioprocess technically and economically unfavorable and it thus cannot be readily translated to the industrial scale.

In this work, we have used the engineered strain of *S. tsukubaensis ΔallR*(18) to develop a chemobiosynthesis process for the efficient and highly selective incorporation of the unnatural extender unit propargylmalonyl-SNAC. We have also developed a method for the efficient synthesis of the precursor propargylmalonyl-SNAC and have optimized an industrially efficient chemobiosynthesis bioprocess for the production of a novel FK506 analogue that contains a terminal alkyne functional group at its C21 position. This technology is potentially applicable to any other enzymatic system of type I PKS, opening a new way for the generation of novel polyketide derivatives containing alkyne functional groups and hence paving the way for “click chemistry” approaches to the development of novel PK natural products.

## Results and Discussion

### Use of the FK506-Producing *ΔallR* Strain, Where the Production of the Allylmalonyl-CoA Extender Unit Is Disrupted

We developed a chemobiosynthetic process where we fed an unnatural α-carboxyacyl-CoA extender unit analogue containing a terminal alkyl moiety propargylmalonyl-SNAC [(*S,S-*bis(2-acetamidoethyl) 2-(prop-2-yn-1-yl) propanebis(thioate)] to cultures of a *S. tsukubaensis* Δ*allR* mutant strain that has an inactivated *allR* gene. Since this gene encodes the crotonyl-CoA carboxylase/reductase, a key enzyme involved in the formation of ethylmalonyl-SCoA and allylmalonyl-SCoA,^18^ these two natural extender units are not biosynthesised in this strain. Therefore, the exclusive biosynthesis of a target FK506 analogue modified at the C21 position can only be achieved in the *S. tsukubaensis* Δ*allR* strain when an unnatural extender unit, fed during the chemo-biosynthetic process, is selected by the AT4 domain.

A few C21-modified FK506 analogues were generated through biosynthetic engineering or feeding procedures in the past.^15^ However, the titer achieved for these metabolites was most often below 1 mg/L, which is not sufficient for thorough semisynthetic work, preclinical evaluations and any potential economical transfer to the industrial scale.^11^

**Figure 2:**
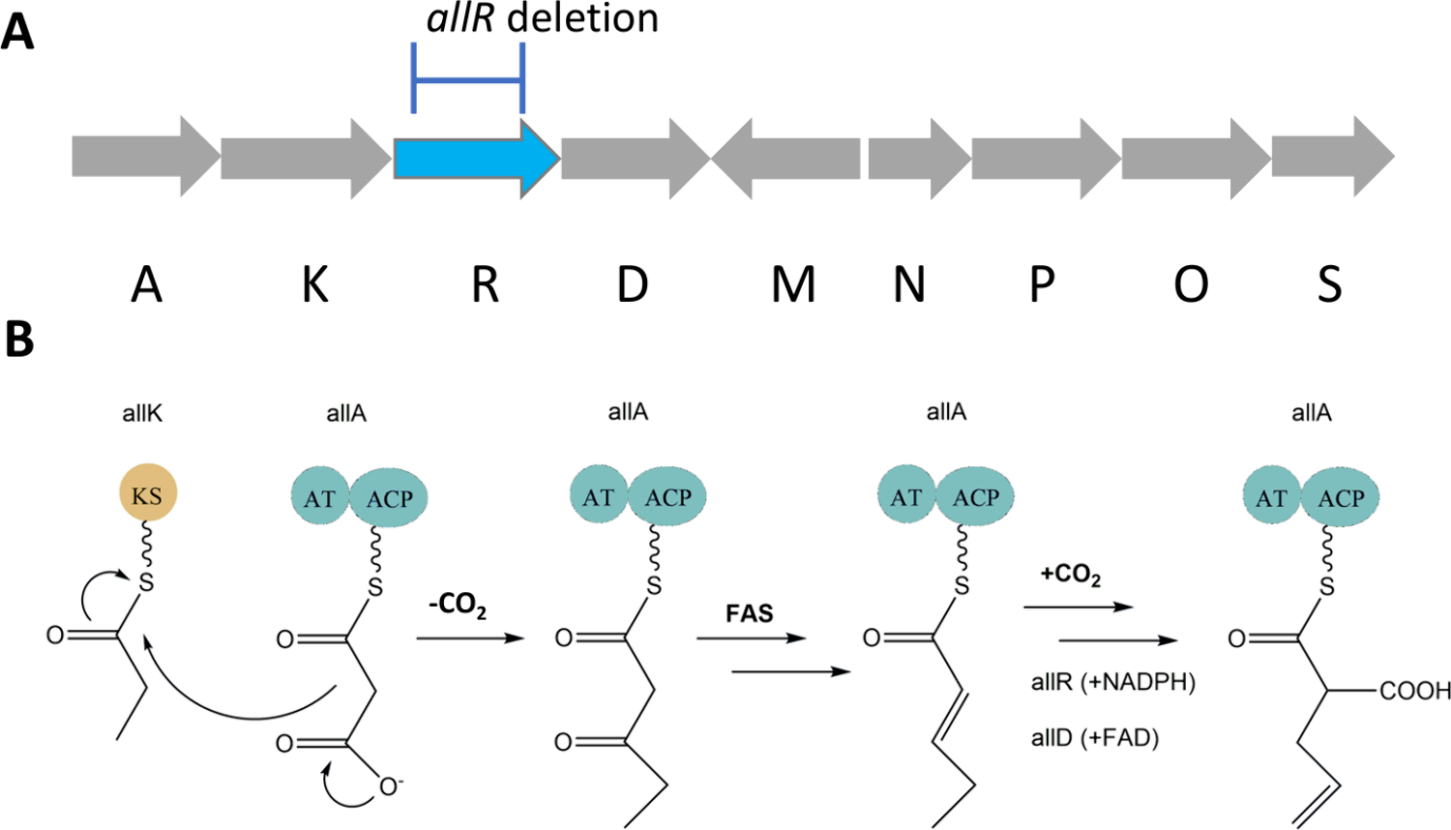
Construction of strain *S. tsukubaensis* Δ*allR*. A) the deletion of *allR* in the FK506 biosynthetic gene cluster disrupts the production of natural allylmalonyl-CoA in FK506, as described by Kosec et al.^18^ The *allA*, *allK*, *allR*, and *allD* (A-K-R-D) gene products, part of the FK506 biosynthetic gene cluster, are involved in the biosynthesis of the allylmalomyl-CoA extender unit, as presented in panel B. B) Proposed biosynthesis pathway of unusual extender unit allylmalonyl used by the AT4 domain of FK506 PKS synthase.^15,16^ Propyl-SCoA is proposed as the starter unit and malonyl-SCoA as the extender unit, selected by the KS and AT domains of AllA, respectively, resulting in the C5 carbon unit after decarboxylative condensation carried out by the KS domain. It seems that no other putative candidate reductase and dehydratase genes involved in the formation of the double bond are present in the FK506 gene cluster. Other ubiquitous enzymes such as fatty acids synthase (FAS) which are encoded in other regions of the chromosome in *S. tsukubaensis* likely carry out these reactions. CCR-homologue 2-pentenoyl-CoA carboxylase/reductase (*allR*) catalyzes the reductive carboxylation of α,β-unsaturated 5 carbon 2-pentenoyl-ACP substrate followed by the final dehydrogenation of acyl-ACP dehydrogenase by AllD, resulting in allylmalonyl-ACP.

Therefore, in the scope of this work, and to ensure sufficient titer of the target product propargyl-FK506, we developed an efficient precursor-directed chemobiosynthetic process with the *S. tsukubaensis* Δ*allR* strain containing an in-frame deletion of *allR*(18) (Figure 2), which produces neither FK506 nor FK520. To achieve this, it was necessary to develop an efficient procedure for the synthesis of the precursor of the unnatural extender unit propargylmalonyl–S-N-acetylcysteamine (propargylmalonyl-SNAC).

### Synthesis of Propargylmalonyl-SNAC [*S,S-*Bis(2-acetamidoethyl)2-(prop-2-yn-1-yl)propanebis(thioate)]

Click chemistry is a powerful tool in the area of synthetic chemistry; it by-passes the need for laborious and often very complex synthetic steps, followed by purification processes, that are characteristic of conventional chemical synthesis.^21,22^ First defined by Nobel laureate Sharpless and associates in 2001, click chemistry consists of a few stereospecific, modular reactions with a high thermodynamic driving force that occur in simple reaction conditions and preferably in an aqueous environment. “Click reactions” often enable high yields of lead compound. For example, among click chemistry reactions, copper(I)-catalyzed azide alkyne cycloaddition is the most used and versatile “click” reaction, which can be used to derivatize the propargyl moiety at the C21 position of FK506.^21,23−25^ The alkyl moiety introduced into the PK scaffold would not only ensure a simple and straightforward reactive moiety at the desired position in the PK backbone, but would also simplify the entire semisynthetic procedure, by avoiding the protection and deprotection steps that are due to undesired off-target reactions.^26^

**Figure 3:**
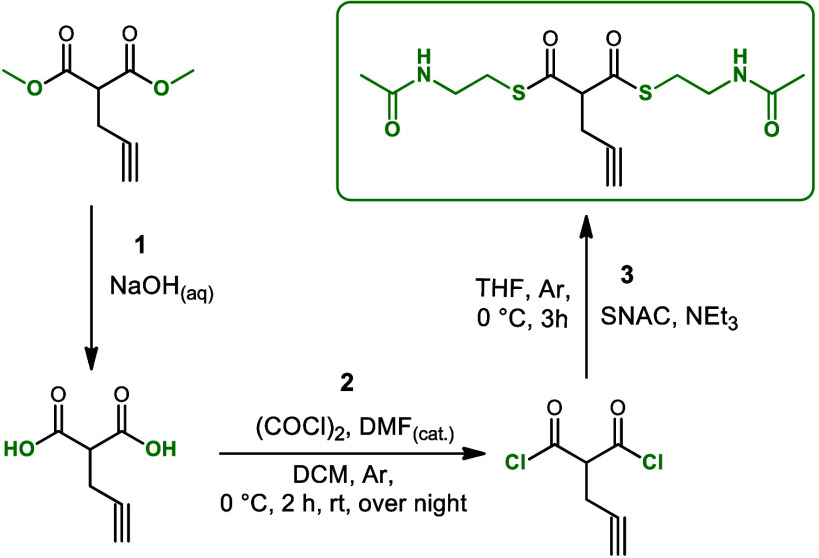
Schematic presentation of the three-step synthesis of propargylmalonyl-SNAC [(*S,S-*bis(2-acetamidoethyl) 2-(prop-2-yn-1-yl)propanebis(thioate)]. The chemical structures of the intermediates and the final target product propargylmalonyl-SNAC [(*S,S-*bis(2-acetamidoethyl)2-(prop-2-yn-1-yl)propanebis(thioate)] were confirmed by ^1^H NMR analysis. Finally, the structure of the final product propargylmalonyl-SNAC was reconfirmed by ^13^C NMR spectra (Figure S4) and MS analysis (Figure S5). The high purity of propargylmalonyl-SNAC was confirmed by HPLC analysis (Figure S6).

We initiated the synthesis of the target extender unit propargylmalonyl-SNAC from dimethyl 2-(prop-2-yn-1-yl)malonate and then hydrolyzed it to a malonic acid derivative by aqueous NaOH (Step1, Figure 3, Figure S1). We further transformed 2-(prop-2-yn-1-yl) malonic acid to the corresponding malonyl chloride (Step 2, Figure 3, Figure S2) that ultimately gave rise to the target compound *S,S-*bis(2-acetamidoethyl) 2-(prop-2-yn-1-yl)propanebis(thioate), by using N-acetylcisteamin (Step 3, Figure 3, Figure S3). The detailed three-step procedure for the synthesis of propargylmalonyl-SNAC (*S,S-*bis(2-acetamidoethyl)2-(prop-2-yn-1-yl)propanebis(thioate)) was carried out as described in Supporting Information (Step 3, Figure 3, Figure S4–S6).

N-Acetylcysteamine (SNAC) thioesters are often used as test surrogates for acyl carrier protein (ACP)-tethered intermediates. In this chemobiosynthetic process, the synthesis of the unnatural extender unit may carry additional costs. SNAC-thioester of propargylmalonyl may not be the most economical version of this activated malonate extender unit. A number of alternative thioesters could instead be generated that replaced SNAC thioesters and became more economical.^27^

### Chemobiosynthesis Process Optimization by Allylmalonyl-SNAC Feeding of *S. tsukubaensis* Wild Type and *S. tsukubaensis ΔallR* Strains

**Figure 4:**
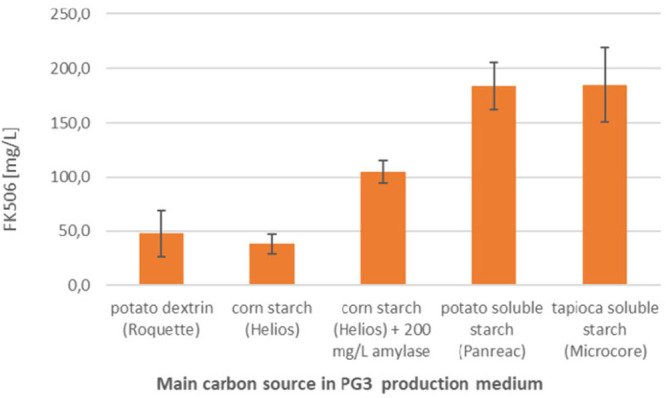
Evaluation of different starch sources as the main carbon source present in PG3 medium for the production of FK506.

Relatively low titers of FK506, from a few milligrams, up to 50 mg/L, are typically achieved with the *S. tsukubaensis* NRRL18448 wild-type strain.^28^ Consequently, even lower titers of an FK506 analogue are achieved when applying a chemobiosynthetic procedure to the engineered strain *S. tsukubaensis ΔallR*, as shown by Kosec et al. (2012).^18^ To improve the efficacy of the chemobiosynthetic process with propargyl-SNAC, we initially carried out medium optimization work for the production of FK506 by testing different carbon sources. As previously reported,^16,18^ FK506 yields were approximately 30–70 mg/L (Experimental Section) when using the *S. tsukubaensis* NRRL18488 wild-type strain in PG3 medium on a shaker scale. Initially, we used dextrin (90 g/L) as the main carbon source in the PG3 production medium. Dextrin was thereafter replaced with several alternative carbon sources, always maintaining the same total carbon concentrations, since the use of alternative starch sources was known to exert a significant impact on FK506 titer.^28^ Thus, starch and soluble starch from various suppliers were tested, and their effect on FK506 production by the *S. tsukubaensis* wild-type strain was evaluated (Figure 4).

We observed a significantly higher titer of FK506 in the production medium that contained soluble starch. Corn starch without amylase pretreatment resulted in FK506 titers that were comparable to those of the PG3 control medium that contained potato dextrin. However, a significant increase in yields resulted from the addition of amylase to PG3 medium containing corn starch, which resulted in a 2-fold increase in FK506 titers, indicating that the starch properties and the degree of hydrolysis had a crucial impact on the final FK506 yields. Potato and tapioca soluble starches were tested as alternatives to avoid the need for amylase addition. Use of soluble starches led to a significant increase in FK506 production, with FK506 titers reaching approximately 200 mg/L at the shake flask-level using the *S. tsukubaensis* NRRL18488 wild-type strain (Figure 4).

The increase in the FK506 yield was likely due to the capability of the producing strain for faster starch assimilation while catabolic repression was avoided by glucose as a result of starch hydrolysis. The PG3 production medium, where dextrin was replaced by soluble starch, was designated as PG3_SS.

### Optimization of the Chemobiosynthetic Process by Feeding Allylmalonyl-SNAC to the *S. tsukubaensis ΔallR* Strain

In the next step, we evaluated different feeding regimes and the quantity of an unnatural extender unit (precursor) to be fed during the process. For this purpose, we used allylmalonyl-SNAC, considering that allylmalonyl-CoA is a native extender unit selected by the AT4 domain.^18^

In our earlier work,^18^ we carried out a chemosynthetic procedure on the FK506-nonproducing *S. tsukubaensis ΔallR* strain, by transferring 33% (v/v) of the production culture after 3 days of cultivation to fresh production medium that already contained allylmalonyl-SNAC. The culture was then cultivated for an additional 6 days.^16,18^ Although we applied the same conditions (i.e., feeding allylmalonyl-SNAC), we obtained significantly lower FK506 titers. Compared to the wild-type strain *S. tsukubaensis* NRRL18448 that achieved titers of 30–70 mg/L, the *ΔallR* strain reached up to 15 mg/L.^18^ As reported in our previous work,^18^ the relatively low titer of the target product was in part likely attributed to toxicity of the unnatural SNAC-thioester of the allylmalonyl extender unit, since the addition of a SNAC-ester to the wild-type strain already resulted in a reduction in the FK506 yield of approximately 50%.^18^

Optimization of the feeding procedure was carried out by adding a 10% solution of allylmalonyl SNAC thioester (w/v), prepared in DMSO, directly to the cultivation broth of the *S. tsukubaensis ΔallR* mutant, to different final concentrations and at various time points during the cultivation procedure (Table 1).

**Table 1:** Optimization of the Chemobiosynthetic Procedure by Applying Different Regimes of Allylmalonyl-SNAC Feeding^a^

	Time of allylmalonyl-SNAC addition to the culture	Addition of allylmalonyl-SNAC in production medium [g/L]	Total concentration of allylmalonyl-SNAC in production medium [g/L]	FK506 yield [mg/L] *S. tsukubaensis* NRRL18448	% FK506 compared to control	FK506 yield [mg/L] *S. tsukubaensis* ΔallR	Mutasynthesis procedure efficiency
1	Control	0.00	0.00	184.19	100%	6.7	0%
2	At inoculation	0.50	0.50	219.77	119%	14.03	6%
3	At inoculation	0.75	0.75	206.21	112%	16.85	8%
4	At inoculation	1.00	1.00	194.96	106%	29.69	15%
5	Day 3	0.50	0.50	208.64	113%	16.21	8%
6	Day 3	0.75	0.75	215.15	117%	26.77	12%
7	Day 3	1.00	1.00	206.47	112%	35.73	17%
8	At inoculation + day 3	0.50	1.00	231.88	126%	28.02	12%
9	At inoculation + day 3	0.75	1.50	193.75	105%	32.23	17%
10	At inoculation + days 1–6	0.50	3.50	179.87	98%	103.01	57%
11	Days 1–6	0.50	3.00	146.42	79%	74.75	51%
12	Days 2–6	0.50	2.50	176.87	96%	62.86	36%
13	Days 3–6	0.50	2.00	164.72	89%	38.80	24%
14	Days 3–6	0.75	3.00	150.14	82%	53.22	35%

a The efficacy of the chemobiosynthetic procedure (last column) is presented as a ratio of the FK506 titer achieved by this procedure compared to the FK506 titer achieved with the parent strain *S. tsukubaensis* NRRL18448 (Figure 5).

Total concentrations of allylmalonyl-SNAC that were added to the culture ranged from 0.5 to 3.5 g/L. The *S. tsukubaensis* wild-type strain was used as the control strain to evaluate the potentially negative effects of the SNAC-thioester on biomass formation. Initial testing of different feeding regimes was carried out in Falcon tubes, containing 5 mL of PG3_SS production medium. Cultivation was carried out at 28 °C for 7 days (Table 1, Figure 5).

**Figure 5:**
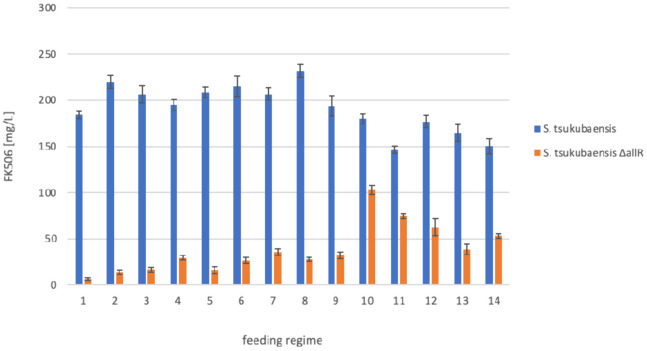
Optimization of the chemobiosynthetic procedure; evaluation of different feeding regimes of allylmalonyl-SNAC on FK506 titers using wild type and the *S. tsukubaensis ΔallR* mutant strain at the 5 mL scale. Feeding regimes under 1–14 are described in Table 1.

Interestingly, the addition at a low total concentration of allylmalonyl-SNAC to wild-type strain *S. tsukubaensis* NRRL18448 showed a positive effect on FK506 yields. When feeding allylmalonyl-SNAC to the engineered strain *S. tsukubaensis ΔallR,* the highest FK506 titer achieved reached up to approximately 50%, compared to the control wild type strain *S. tsukubaensis* NRRL18448 (Table 1 and Figure 5, regimes 1–14). This increase in FK506 titer was observed, while the total amount of added allylmalonyl-SNAC did not exceed 1 g/L. Clearly, the increase in FK506 titer in the WT strain *S. tsukubaensis* NRRL18448 indicates that a natural supply of the allylmalonyl-CoA extender unit is a limiting factor in the biosynthesis of FK506. The feeding regimes where the total amount of SNAC-thioester added to the culture exceeded 1.5 g/L resulted in a decreased FK506 titer when using wild-type strain *S. tsukubaensis* NRRL18448, indicating that higher concentrations of the unnatural extender unit allylmalonyl-SNAC dissolved in DMSO are toxic to the culture (Table 1 and Figure 5, regimes 10–14).

**Figure 6:**
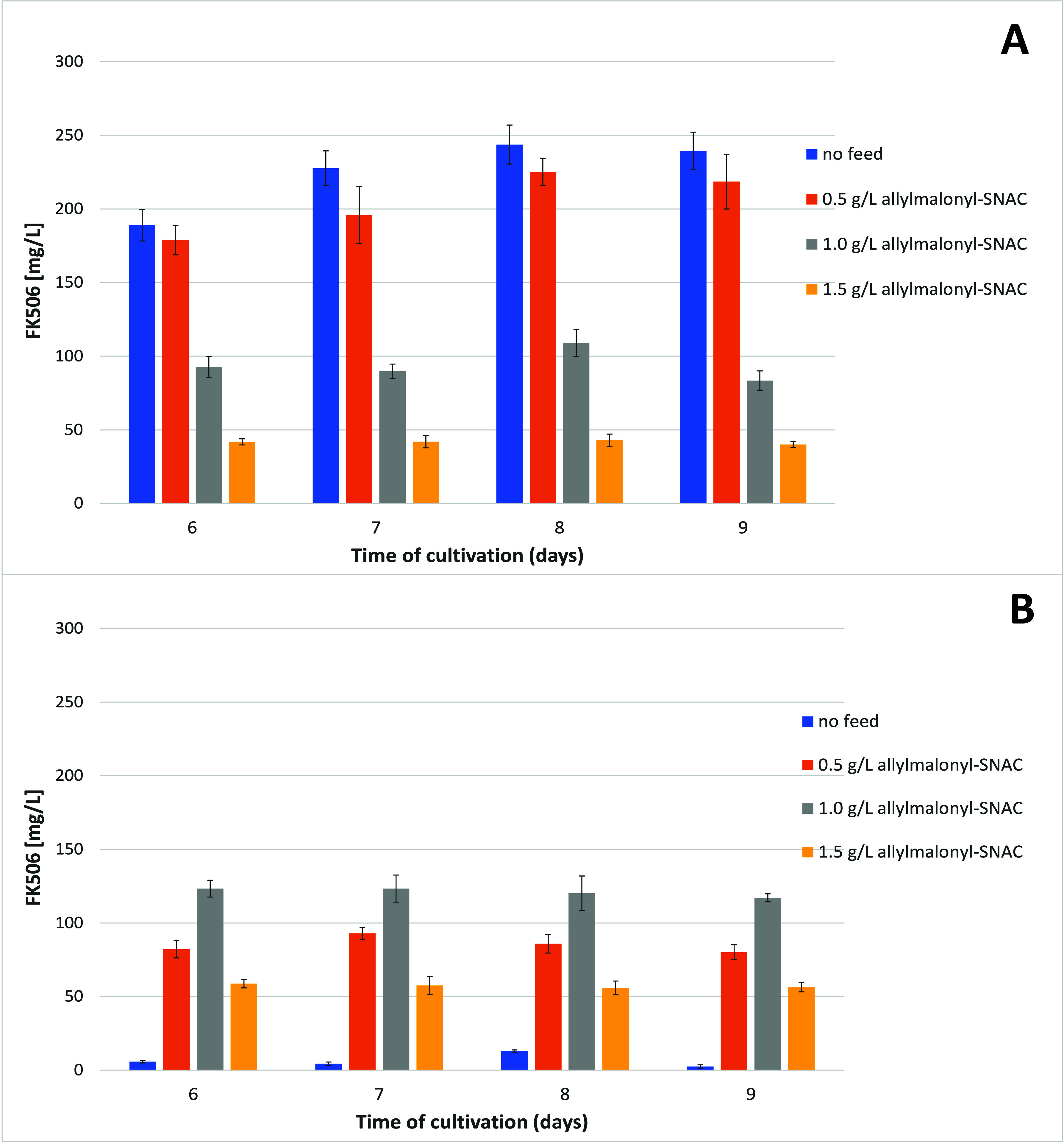
Chemobiosynthetic process in 250 mL flasks. Addition of allylmalonyl-SNAC was carried out at inoculation and every 24 h thereafter until the 6th day of cultivation. Cultivation was then carried out for an additional 3 days in the absence of any additional feeding and to a total of 9 days; A) wild-type *S. tsukubaensis* NRRL18448 control; B) *ΔallR* mutant strain.

The effect of different feeding regimes on the final FK506 titer was particularly pronounced with the *S. tsukubaensis ΔallR* strain. A comparative correlation of the FK506 titers achieved with *S. tsukubaensis ΔallR* and the wild type strains (Figure 6), simultaneously cultivated in identical cultivation media and the same allylmalonyl-SNAC feeding procedure, was regarded as an indicator of the efficacy of the allylmalonyl-SNAC thioester feeding procedure.

The highest titer of FK506 achieved when applying the chemobiosynthetic procedure, feeding allylmalonyl-SNAC thioester to the *S. tsukubaensis ΔallR* strain, was approximately 100 mg/L. This titer was obtained when a maximum total amount of 3.0 g/L allylmalonyl-SNAC was added to the culture broth at 7 time points during cultivation (Table 1, regime #10). At each time point, the allylmalonyl-SNAC ester was fed to the culture broth to a final concentration of 0.5 g/L, starting at the time of inoculation and then repeating the feeding procedure every 24 h during the six-day cultivation procedure. Thus, the total concentration of allylmalonyl-SNAC ester was 3.0 g/L.

The most efficient chemobiosynthetic procedure at the 5 mL scale was subsequently tested in shake flasks, and the FK506 yield was determined after 6–9 days of cultivation, in order to determine the optimal cultivation time. Allylmalonyl-SNAC thioester was added to the production medium at concentrations of 0.5–1.5 g/L, at the time of inoculation and thereafter continuing with the same feeding rate every 24 h during 7 days of cultivation. Thus, from 4 g/L to a maximum of 12 g/L of total allylmalonyl-SNAC thioester had been fed to the culture by the time the last feeding had been carried out (Figure 6).

The results from the chemobiosynthetic procedure in shake flasks were in good correlation with the results obtained at the 5 mL scale (Figure 6B). When allylmalonyl-SNAC feeding was carried out to *S. tsukubaensis ΔallR* cultures to a final concentration of 0.5 g/L at each time-point, approximately 90 mg/L FK506 was achieved after 7–8 days of cultivation. At the lowest total concentration of 0.5 g/L allylmalonyl-SNAC-thioester feeding, we observed a minor negative effect on FK506 titer in the wild-type strain (up to 15% reduction). However, the FK506 titer obtained with the WT strain *S. tsukubaensis* NRRL18448 at the highest total feeding concentrations of 1.0 and 1.5 g/L was significantly reduced to approximately 50–65% and 80–85%, respectively. Partial cell volume (PCV) was measured to determine any potential effect of SNAC-thioester feeding on the formation of biomass following 9 days of cultivation. PCV measurements (Table S1) indicated that the addition of allylmalonyl-SNAC had an inhibitory effect on biomass formation and pH value, which resulted in a reduction in FK506 biosynthesis by the wild-type strain. Based on the data gathered with the *S. tsukubaensis* Δ*allR* strain, where allylmalonyl-SNAC feeding was carried out, we can conclude that feeding 0.5 g/L SNAC-thioester at each time-point was the most efficient and economical approach. Moreover, although the *S. tsukubaensis* Δ*allR* mutant strain was used, the optimized feeding procedure achieved a relatively high titer of around 100 mg/L of FK506, compared to the native strain. Importantly, we have demonstrated that even without the auxiliary gene *allR* (Figure 2) being involved in the synthesis and provision of native allylmalonyl-CoA, the corresponding AT4 domain was able to efficiently select for the allylmalonyl-SNAC precursor.

### Development of a Chemobiosynthetic Process for the Production of C21 Propargyl-FK506

**Figure 7:**
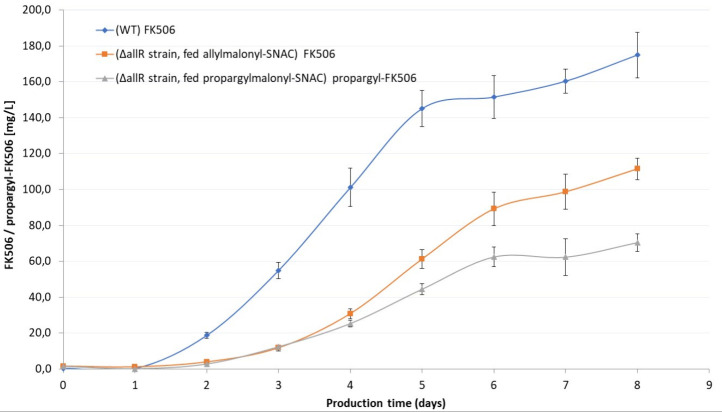
Chemobiosynthetic process for the production of FK506 and propargyl-FK506 in shake flasks. Allylmalonyl-SNAC and propargylmalonyl-SNAC (10% w/v solutions in DMSO) were added to the production medium at the time of inoculation and every 24 h thereafter, up to 7 days.

The chemobiosynthetic process for the production of propargyl-FK506 was carried out by feeding the propargylmalonyl-SNAC extender unit (10% w/v in DMSO) to the *S. tsukubaensis* Δ*allR* strain. We applied the chemobiosynthetic procedure according to the optimized procedure described above, where 0.5 g/L propargylmalonyl-SNAC was fed to the production medium every 24 h up to 7 days (Figure 7). As presented in Figure 7, when feeding the unnatural extender unit propargylmalonyl-SNAC, the chemobiosynthetic process was slightly less efficient compared to the process with allylmalonyl-SNAC. The likely reason is that the AT4 domain has lower affinity for propargylmalonyl-SNAC than for allylmalonyl-CoA. Nevertheless, a final yield of around 70 mg/L of propargylmalonyl-FK506 was achieved, thus easily ensuring sufficient amounts of the target compound. The HPLC analysis of crude material and of the purified propargyl-FK506 analogue is presented in Figures S7 and S8, respectively. LC-MS analysis of the final product isolated by preparative HPLC is presented in Figure S9.

AT-swap in PKS systems has been a successfully employed technology for two decades. For example, Del Vecchio et al.^13^ have demonstrated that the AT4 domain from the erythromycin PKS module 4 can be successfully replaced with the AT2 domain from the rapamycin PKS module 2 to alter its specificity from methylmalonyl-CoA to malonyl-CoA.

It is not yet fully understood how the FK506 PKS complex, in collaboration with an auxiliar biosynthetic complex, transfers an allylmalonyl-CoA extender unit to the AT4 domain of the FkbB protein of FK506 PKS. Goranovič et al.^16^ and Mo et al.^15^ demonstrated that allylmalonyl-CoA is synthesized by an unusual small protein complex containing KS-AT-ACP domains encoded by a small gene cluster (*allA, allK, allR* and *allD*), located on the side of the FK506 biosynthetic gene cluster (Figure 2). Experiments by Jiang et al. suggest that both ACP and CoA can be acyl donor candidates for the transfer of an allylmalonyl unit catalyzed by AT4 from the FK506 PKS.^29^ Therefore, it is possible that any type I PKS complex containing a heterologous AT4 domain from the FkbB module 4 of FK506 PKS could potentially accept an unnatural propargylmalonyl extender unit and thus result in a polyketide backbone containing a propargyl moiety in the presence of propargylmalonyl-SNAC. Alternatively, to ensure a more efficient transfer of the unnatural extender unit propargylmalonyl-SNAC, auxiliary genes for the provision of allylmalonyl-CoA should be used in a heterologous host together with a type I PKS complex containing a heterologous AT4 domain from the FkbB module 4 of FK506 PKS. If successful, this approach would enormously expand our capability to derivatize numerous PKs of medical and industrial importance.

In this work, we have established a reproducible production method and industrially relevant titer of the target compound, and we therefore easily prepared sufficient amounts of pure propargyl-FK506 at the shake-flask level.

### Structure Confirmation of C21 Propargyl-FK506

The elucidation of the structure of C21 propargyl-FK506 was achieved through one- and two-dimensional NMR experiments. ^1^H and ^13^C NMR spectra of a C21 propargyl-FK506 sample showed two sets of signals in a ratio 2:1 (Figures S10–S11 and S16); these were attributed to two conformational isomers (rotamers) arising from the restricted rotation of the amide bond. The starting point for the ^1^H and ^13^C NMR assignment was a propargyl group attached to C21, which showed characteristic chemical shifts for C/H38–C/H40. ^1^H–^1^H (Figures S12–S13) and ^1^H–^13^C correlation signals via single and multiple bonds (Figures S14–S15) enabled the unequivocal assignment of signals on the macrolactam ring. The ^1^H–^13^C gHMBC spectrum (Figure S15) showed correlation signals to ketone carbons C9 (δ_*C*_ = 199.40 and 200.09 ppm) and C22 (δ_*C*_ = 210.13 and 209.38 ppm). C8 showed characteristic chemical shifts of the amide group (δ_*C*_ = 167.47 and 167.70 ppm). The carbon atom C1 of the ester group (δ_*C*_ 170.42 and 170.50 ppm) was confirmed by multiple bond correlation signals with both the H2 and H26 protons. Two sets of signals were observed in the ^1^H and ^13^C NMR spectra, which were attributed to the *cis* and *trans* rotamers along the peptide bond. The ratio between the *cis* and *trans* rotamers was 2:1 for C21 propargyl-FK506. ^1^H and ^13^C NMR chemical shifts for both rotamers of C21 propargyl-FK506 are presented in Table 2. The chemical structure is in full agreement with the C21 propargyl-FK506 structure presented in Figure 1. *Cis* and *trans* rotamers along the amide bond can be distinguished with respect to the characteristic differences in chemical shifts of C2 and C6 (δ_*C*_ 57.5 and 39.86 ppm for the *cis* rotamer; δ_*C*_ 53.36 and 45.01 ppm for the *trans* rotamer).

**Table 2:** NMR Spectroscopic Data (600 MHz, Pyridine-*d*_5_) for Propargyl-FK506

	Major rotamer (*cis*)^a^		Minor rotamer (*trans*)^a^	
Position	δ_*C*_, type	δ_*H*_ (*J* in Hz)	δ_*C*_, type	δ_*H*_ (*J* in Hz)
1	170.42, C	–	170.50, C	–
2	57.5, CH	5.16, m	53.36, CH	5.59, d (5.4)
3	/b	/b	/b	/b
4	21.97, CH_2_	1.6–1.7, m, 1.7–1.8, m	22.32, CH_2_	1.5–1.6, m, 1.6–1.7, m
5	25.27, CH_2_	1.46, m, 1.62, m	25.64, CH_2_	1.38, m, 1.55, m
6	39.86, CH_2_	3.29, m, 4.75, m	45.01, CH_2_	3.6–3.7, m
8	167.47, C	–	167.70, C	–
9	199.40, C	–	200.09, C	–
10	99.40, C	–	100.13, C	–
11	36.33, CH	2.60, m	35.9, CH	2.80, m
12	33.49, CH_2_	1.82, m, 2.22, m	33.32, CH_2_	1.89, m, 2.22, m
13	74.73, CH	3.68–3.74, m	75.15, CH	3.68–3.74, m
14	73.48, CH	4.24, d (9.6)	74.41, CH	4.31, dd (9.6, 2.9)
15	76.66. CH	3.90, m	78.40, CH	3.90, m
16	34.99, CH_2_	1.47, m, 1.79, m	35.57, CH_2_	1.65, m, /b
17	26.93, CH	1.98, m	27.0–27.2, CH	2.05, m
18	49.57, CH_2_	2.0–2.1, m	48.09, CH_2_	2.01, m, /b
19	140.59, C	–	141.48, C	–
20	123.16, CH	5.16, m	123.38, CH	5.20, d (10.1)
21	53.20, CH	3.99, dt (10.2, 7.0)	53.09, CH	4.05, dt (10.0, 7.2)
22	210.13, C	–	209.38, C	–
23	48.31, CH_2_	2.74, dd (14.4, 6.4), 3.17, m	46.75, CH_2_	3.00, dd (16.3, 7.6), 3.09, dd (16.3, 4.8)
24	69.81, CH	4.56, m	69.24, CH	4.65, m
25	41.85, CH	2.12, m	41.65, CH	2.25, m
26	81.34, CH	5.87, d (5.8)	79.93, CH	5.93, d (5.0)
27	132.80, C	–	133.29, C	–
28	133.76, CH	5.52, d (8.9)	132.56, CH	5.50, d (9.0)
29	35.9, CH	2.44, m	35.9, CH	2.44, m
30	36.81, CH_2_	1.15, m, 2.17, m	36.83, CH_2_	1.15, m, 2.17, m
31	85.44, CH	3.24, m	85.50, CH	3.24, m
32	74.24, CH	3.71, m	74.26, CH	3.70, m
33	33.88, CH_2_	1.61, m, 2.11, m	33.89, CH_2_	1.61, m, 2.11, m
34	31.66, CH_2_	1.0–1.1, m, 1.5–1.7, m	31.69, CH_2_	1.0–1.1, m, 1.5–1.7, m
35	17.30, CH_3_	1.30, d (6.5)	16.7, CH_3_	1.21, d (6.8)
36	20.30, CH_3_	0.97, d (6.5)	20.9, CH_3_	0.98, d (6.3)
37	16.7, CH_3_	1.69, s	17.44, CH_3_	1.90, s
38	21.47, CH_2_	2.54, m, 2.76, m	20.9, CH_2_	2.62, m, 2.84, m
39	83.51, C	–	83.78, C	–
40	71.24, CH	2.70, t (2.5)	71.25, CH	2.72, t (2.6)
41	11.26, CH_3_	1.23, d (6.8)	11.05, CH_3_	1.19, d (7.0)
42	13.75, CH_3_	1.84, s	14.12, CH_3_	1.90, s
43	56.55, CH_3_	3.43, s	56.27, CH_3_	3.44, s
44	57.94, CH_3_	3.46, s	58.11, CH_3_	3.47, s
45	57.56, CH_3_	3.47, s	57.53, CH_3_	3.46, s

a Ratio between *cis* and *trans* rotamers is 2:1.

b NMR chemical shifts could not be unequivocally assigned due to significant signal overlap.

### Evaluation of the Biological Activity of C21 Propargyl-FK506

**Figure 8:**
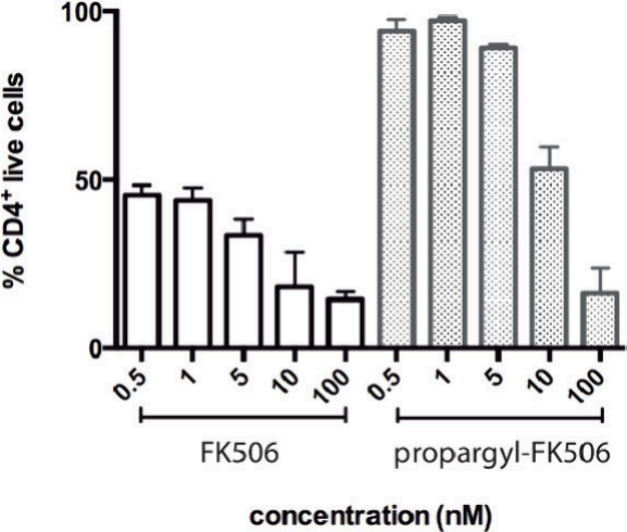
Cytotoxicity of FK506 and C21 propargyl-FK506. After 4 days of culture, CD4^+^ live cells were analyzed by flow cytometry (Figure S18). Bar graphs represent the frequency of live cells normalized to nonstimulated control samples.

In the scope of this work, we isolated sufficient amounts of propargyl-FK506 to determine its structure (Supporting Information) and evaluate its activity. In order to compare the toxicity of FK506 and its C21 propargyl analogue, we established primary T cell cultures (DC Balb/c, T cells C57Bl/6) and exposed them to increasing concentrations of FK506 and propargyl-FK506. Rates of live cells were evaluated by staining for CD4^+^ and LIVE/DEAD Fixable Aqua, following a 4-day treatment; frequencies were normalized to nonstimulated live cells (Figure 8, Figure S18). High cell death rates were observed when testing FK506 at different concentrations (0.5 1, 5, 10, and 100 nM). However, a toxic effect of C21 propargyl-FK506 was observed only when this analogue was applied at concentrations higher than 10 nM.

**Figure 9:**
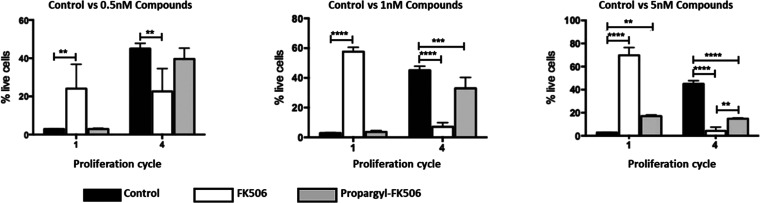
Immunosuppressive activity of FK506 and C21 propargyl-FK506 at 0.5, 1, and 5 nM concentrations. Naïve CD4^+^ T-cells derived from C57BL/6 mice were cocultured with allogeneic bone marrow-derived BALB/c DCs, and incubated with 0.5, 1, and 5 nM concentrations of either tacrolimus or propargyl-FK506, in order to compare the immunosuppressive potential of these two compounds. Representative flow cytometry results showing the rates of live CD4^+^ T cells at proliferation cycles 1 and 4 under each of the conditions: control and treated with 3 different concentrations of each of the compounds. The proliferation was assessed by flow cytometry (Figure S19). The gating strategy is presented in Figure S20. The data shown here and Figure S19 are the combined results from three independent experiments. Graphs show mean values, and error bars represent SDs unless otherwise specified. *P < 0.05; **P < 0.01; ***P < 0.001; ****P < 0.0001.

To investigate the immunosuppressive potential of the C21 propargyl-FK506 analogue, we compared the expansion of alloreactive effector T cells that were either exposed to this compound or to FK506 while grown for 4 days in coculture with Dendritic cells (DCs) derived from BALB/c mice; proliferation was evaluated via CellTrace Violet stain incorporation. As expected, FK506 exhibited strong immunosuppressive activity and could inhibit cell proliferation at 1 nM (Figure 9). Gating strategies are presented in Figure S20. Immunosuppression by C21 propargyl-FK506 was detected at 5 nM (Figure 9), with greatly reduced cell numbers at cell cycle 4 (Figure 9); yet, cell viability was sustained at this concentration (Figure 8). Thus, it can be concluded that C21 propargyl-FK506 is less toxic to primary T cell cultures than FK506, while exerting its immunosuppressive effect at higher concentrations.

Interestingly, although allyl and propargyl moieties only marginally differ in their structure (terminal triple bond instead of a double bond), the replacement of the allyl moiety with the propargyl-moiety did have a profound effect on the immunosuppressive activity and toxicity of the novel compound. Propragyl-FK506 displays significantly reduced immunosuppressive activity while being significantly less toxic to primary T cell cultures.

Nevertheless, this FK506 analogue still preserves some immunosuppressive effect and could thus be used for further diversification of the terminal alkyl group of the propargyl-moiety at carbon C21 of the FK506 backbone. As exemplified in the literature, FK506 analogues with reduced immunosuppressive activity and toxicity, and increased antifungal or nerve-regenerating activities are of interest in the scope of drug-development efforts.^10,30^

## Experimental Section

### General Experimental Procedures

NMR spectra were acquired on an Agilent Technologies 800 MHz NMR spectrometer equipped with a cryoprobe and operating at 800 MHz for ^1^H and at 200 MHz for ^13^C nuclei. All NMR data were processed using Mnova software (Mestrelab Research S.L.). Detection of FK506 and analogues were carried by Nucleosil EC100–3 C_18_, reversed-phase HPLC column. The mobile phase used for isocratic elution was composed of water, acetonitrile, MTBE and phosphoric acid (58.29:34.4:7.29:0.02, v/v/v/v). Isolation of propargyl-DK506 was carried out by an extraction procedure followed by preparative HPLC purification using Knauer preparative HPLC with a Macherey-Nagel C_18_ column as stationary phase. LC-MS/MS analyses were performed on an Agilent 1100 with a reversed phase analytical C18 column (Gemini C18 column, 5 μm, 150 mm × 2 mm i.d., Phenomenex, Torrance, CA, USA). The mass selective detector (Quattro Micro API, Waters, Milford, MA, USA) equipped with an electrospray ionization, and cone voltage of 20 V and capillary voltage of 3.0 kV were used for positive ionization of the analytes.

### Cultivation of Engineered Cultures

The *S. tsukubaensis* NRRL 18448 strain was used for optimization of the production medium. The FK506 nonproducing *S. tsukubaensis* Δ*allR* mutant strain with an inactivated *allR* gene (crotonyl CoA carboxylase in the FK506 gene cluster) was used for chemo-biosynthetic bioprocess experiments.^16,18^ For the preparation of spore stocks, the *S. tsukubaensis* strains were cultivated as a confluent lawn on ISP4 agar sporulation medium^31^ for 10–14 days at 28 °C. For liquid cultures, spores of the *S. tsukubaensis* strains were inoculated in VG3 seed medium^16^ and incubated at 28 °C and 250 rpm for 24–48 h. This seed culture (10% v/v) was then used to inoculate 250 mL Erlenmeyer flasks containing 50 mL of production medium based on the PG3 production medium recipe, as described previously.^16^ Cultivation was carried out at 28 °C and 250 rpm for 6–7 days. Different sources of starch were used as a main carbon source in the PG3 medium, as described in the Results and Discussion. Partial degradation of the starch was carried out by the addition of alpha-amylase (Glentham life sciences, GE7409) to the PG3 medium. The effect of SNAC-thioesters on biomass formation was evaluated by the packed cell volume (PCV). For the determination of PCV, 10 mL of culture broth was transferred to a 15 mL Falcon tube and centrifuged at 4000 rpm for 10 min. The volume of the sediment, which contained the *S. tsukubaensis* biomass, was then recorded.

### Detection of FK506 and Propargyl-FK506

The analytical procedures used in this work have been previously described.^16^ Briefly, after 6–7 days of cultivation, the broth was mixed with an equal volume of methanol (1:1) and the soluble fraction loaded onto a Nucleosil EC100–3 C_18_, reversed-phase HPLC column. The mobile phase used for isocratic elution was composed of water, acetonitrile (MeCN), methyl *tert*-butyl ether (MTBE) and phosphoric acid (H_3_PO_4_) (58.29:34.4:7.29:0.02, V/V/V/V). Chromatographic peaks corresponding to FK506 and FK520 were identified using an FK506 and FK520 external standard (obtained from Lek/Sandoz) and ChromQuest software, which was used for data analysis. Using this method, the detection limit for FK506 and FK520 was 0.5 mg/L.

### Statistical Analysis of FK506-Related Metabolites

When the M4018 transformants were compared with each other, statistical analysis was performed using analysis of variance (ANOVA), with SPSS windows version 26.0 (SPSS Inc., Chicago, IL, USA). Mann–Whitney U-tests were used for the comparisons of the FK506 titers from the parental strain and their transformants, at the level of *P* < 0.05.^32^

### Molecular Biology Methods

In this study, the *S. tsukubaensis* Δ*allR* strain with an inactivated *allR* gene was used, as described previously.^18^ Briefly, Goranovič et al.^16^ identified a group of genes encoding the biosynthesis of the extender unit (allylmalonyl-CoA) that forms the allyl group at carbon C21 of FK506 (Figure 1). This group of genes contains a small independent diketide synthase *alllK* system involved in the biosynthesis of the allyl group. Based on this finding, Goranovič et al.^16^ proposed a biosynthetic pathway for the provision of an unusual five-carbon extender unit allylmalonyl-CoA, which is carried out by a novel diketide synthase complex. This small group of genes also contains the *allR* gene (Figure 2), which plays an important role in the provision of the allyl group at carbon C21 of FK506 (Figure 1). *allR* gene is a homologue of the crotonyl-CoA carboxylase/reductase that catalyzes the carboxylation and reduction of crotonyl-ACP toward 2-pentenoyl-ACP, which is an important step in the biosynthesis of the unusual extender unit allylmalonyl-ACP, as described by Goranovič et al.^16^ Based on this information, Kosec et al.^18^ constructed the strain with carrying an inactivated *allR* gene, and developed an efficient chemobiosynthetic process. The main advantage of this strain is that it cannot produce FK506 or any other FK506-related product. Only after feeding synthetically prepared precursors such as allylmalonyl-SNAC is the biosynthesis of FK506 reestablished at a very high efficiency. Therefore, this chemobiosynthetic bioprocess ensures the exclusive production of target FK506-related products, which represents an industrially important advantage.

### Synthesis of Allylmalonyl-SNAC and Propargylmalonyl-SNAC

Synthesis of allylmalonyl-SNAC [*S,S*-bis(2-acetamidoethyl)2-allylpropanbis(thiolate)] was carried out as described by Kosec et al.^18^ The synthesis of [*S,S-*bis(2-acetamidoethyl)2-(prop-2-yn-1-yl)propanebis(thioate)], which contains a terminal alkyne (also designated as propargylmalonyl-SNAC), was carried out for the first time during this work, and it is therefore described in the Results and Discussion.

### Isolation of FK506 Analogues

Around 1000 mL of a fermentation broth culture of *S. tsukubaensis* 2-(prop-2-yn-1-yl) malonic acid-containing C21 propargyl-FK506 was obtained. Fresh broth was subjected to downstream processing by the following procedure: Whole broth was mixed with 1 L of 2-propanol and the mixture was stirred vigorously at room temperature (RT) for 1 h. The suspension was centrifuged at 4500 rpm for 10 min, resulting in 1850 mL of clear supernatant and 157 g of dry biomass. Both fractions were analyzed by HPLC for propargyl-FK506 content. The supernatant was concentrated under reduced pressure on a rotary evaporator to a final volume of 500 mL. The resulting aqueous concentrate was subjected to L/L extraction using equivolume amounts of toluene. The extraction was carried out at RT and was repeated twice. The phases were separated in a separating funnel and both phases were analyzed by HPLC for propargyl-FK506 content. The organic extract was concentrated to give 538 mg of an orange oily slurry. The crude material was subjected to further purification using normal phase silica gel column chromatography. The mobile phase consisted of dichloromethane (DCM) and MeOH, starting with pure DCM and increasing the MeOH share by 10% in each subsequent fraction up to a final MeOH share of 50%. 500 mL (2 volumes) of mobile phase was used for each subsequent elution. The eluates were analyzed for FK506 content by HPLC and it was observed that most of the FK506 was eluted within the mobile phase with a 20% MeOH. Following the evaporation of the solvent, 300 mg of orange-brown material was obtained and subjected to purification by preparative HPLC. Preparative HPLC purification was carried out using Knauer preparative HPLC with a Macherey-Nagel C_18_ column (dimensions 18 mm × 5 mm, particle size: 5 μm) as stationary phase. The mobile phase consisted of a two-solvent system: (A) 60% H_2_O, 33% MeCN and 7% MTBE with 2 ‰ H_3_PO_4_ and (B): 40% H_2_O, 50% MeCN and 10% MTBE with 2 ‰ H_3_PO_4_. The initial composition of the mobile phase was 50% A - 50% B and the gradient was increased over 32 min to 100% B. The flow rate was 12 mL/min and the load was 50 mg of crude material. The sample was purified twice, and the fractions containing a single peak at RT= 22.2 min were collected and subjected to isolation. Isolation was carried out by L/L extraction using MTBE as the organic solvent. The organic phase was dried over anhydrous Na_2_SO_4_ and concentrated to yield 70 mg of white crystalline material. HPLC analysis of the final product, a typical preparative HPLC chromatogram, and the LC-MS analysis are shown below in Figures S7, S8 and S9. The final material was dried overnight under vacuum to give a final amount of 64 mg and it was used for two-dimensional NMR experiments directed at elucidating the structure of the compounds.

### Confirmation of the C21 Propargyl-FK506 Structure

Samples were dissolved in deuterated pyridine (Pyridine-d_5_). 1D (^1^H NMR, ^13^C NMR) and 2D (^1^H–^1^H COSY, ^1^H–^1^H TOCSY, ^1^H–^13^C HSQC, and ^1^H–^13^C HMBC) NMR spectra were acquired on an Agilent Technologies 800 MHz NMR spectrometer equipped with cryo-probe and operating at 800 MHz for ^1^H and at 200 MHz for ^13^C nuclei. The temperature for the samples was set at 298 K. ^1^H and ^13^C NMR chemical shifts were reported in parts per million and referenced with respect to the residual solvent signals, corresponding to TMS at δ = 0.0 ppm. All NMR data were processed using Mnova software (Mestrelab Research S.L.).

In addition, we reconfirmed the structure of C21 propargyl-FK506 by applying LC-MS/MS analysis (Table S2), as described in detail in Supporting Information (6. Additional structural confirmation of C21 propargyl-FK506 by LC-MS/MS analysis).^33^

### Evaluation of the Immunosuppressive Activity of C21 Propargyl-FK506

All mice were maintained under specific pathogen-free conditions at the animal facility at TWINCORE (Hannover, Germany). All animal procedures, such as organ collection, were performed in compliance with the German animal protection law and were approved by the Lower Saxony Committee on the Ethics of Animal Experiments as well as the responsible state office (Lower Saxony State Office of Consumer Protection and Food Safety under permit number 33.9–42502–04–15/1851).

### Mouse T Cell Cultures

CD4^+^CD25^–^ T cells were enriched from the spleen and lymph nodes of C57BL/6 wild type male mice using a Mouse CD4+ T Cell Isolation Kit (Stem Cell Technologies, Canada) and incubated with anti-CD25-PE antibody (eBioscience, USA). Cells were then passed through magnetic columns. This protocol yielded an average of 90% pure CD4+ T cells. T helper cultures were kept for 4 days in IMDM GlutaMAX medium (Life Technologies, USA) supplemented with 10% fetal calf serum (FCS, Biochrom, UK), 500 U penicillin-streptomycin (PAA laboratories, Canada) and 50 μM β-mercaptoethanol (Life Technologies, USA). At day 0, 2–3 × 10^5^ naïve T cells were cultured per well, in the presence of plate-bound anti-CD3ε (10 ug mL^–1^, clone 145–2C11; Bio X Cell, USA), anti-CD28 (1ug/mL, clone 37.51; Bio X Cell, USA), anti-TGF-β1 (2 ng mL-1; Peprotech, USA), rmIL-6 (5 ng mL-1; Peprotech, USA) and rmIL-1β (50 ng mL-1; Peprotech, USA).

### *In Vitro* Allogeneic T-Cell Stimulation

CD4^+^CD25^–^ T cells were enriched from C57BL/6 wild type male mice using a Mouse CD4+ T Cell Isolation Kit (Stem Cell Technologies, Canada) and incubated with anti-CD25-PE antibody (eBioscience, USA). Cells were then passed through magnetic columns. This protocol yielded an average of 90% pure CD4+ T cells. 1.5 × 10^5^ T cells labeled with CellTrace Violet Cell Proliferation (Life Technologies, USA) were cocultured with 5 × 10^4^ GM-CSF bone-marrow-derived allogeneic DCs from BALB/c mice. The coculture was kept for 4 days in RPMI 1640 GlutaMAX medium (Life Technologies, USA) supplemented with 10% heat-inactivated FCS (Biochrom, Germany), 500 U penicillin-streptomycin (PAA laboratories, Canada) and 50 μM β-mercaptoethanol, in the presence or absence of the compounds FK506 and C21 propargyl-FK506. The statistical parameters applied can be found in the figure legends. Data were analyzed by using GraphPad Prism 7.0 software (GraphPad Software, La Jolla, Calif). Statistical analyses were performed as follows: two-way ANOVA followed by Sidak multiple comparison was used to analyze experiments with 2 variables and 3 or more groups, and one-way ANOVA followed by Duvett comparison with a control was used for experiments with 1 variable and 3 or more groups. The experiments with 2 groups were analyzed with the Student *t* test. In all cases, *P* < 0.05 was considered statistically significant.

### Flow Cytometry and Antibodies

Following their isolation from spleen and peripheral lymph nodes, lymphocyte cell suspensions were incubated with Fc Block (clone 2.4G2) for 5 min before staining. Clones and suppliers of mAbs and reagents were: anti-CD4 PE (GK1.5) and anti-CD44 FITC (IM7), from eBioscience (USA); Aqua reagent for live/dead discrimination, from BioLegend (USA); and CellTrace Violet Cell Proliferation kit, from Life Technologies. Cytometric data were acquired on a CyAn ADP (Beckman Coulter, USA) and analyzed with FlowJo Software (Treestar, Ashland, OR, USA).

## Data Availability

The NMR data for propargyl-FK506 has been deposited in the Natural Products Magnetic Resonance Database (NP- MRD; www.np-mrd.org) and can be found at NP0332700 (10.57994/1960).

## Supporting Information Available

^1^H NMR, ^13^C NMR and MS spectra for all synthesized or isolated compounds, ^1^H NMR, ^13^C NMR, ^1^H–^1^H gCOSY NMR, and ^1^H–^1^H TOCSY NMR spectra; ^1^H–^13^C gHSQC NMR, ^1^H–^13^C gHMBC NMR, ^13^C NMR of isolated compound; additional experimental details, including information on feeding regimes of the packed cell volume and pH value of cultures of *S. tsukubaensis* strains and T cell proliferation assays (PDF)
