# Supplementary material for: Efficient and Selective Biosynthesis of a Precursor-Directed FK506 Analogue: Paving the Way for Click Chemistry
Source: J Nat Prod. 2025 Mar 10;88(3):619–30. doi: 10.1021/acs.jnatprod.4c00394 (PMC11959593; doi:10.1021/acs.jnatprod.4c00394)
Supplement: Supplementary file 1 — np4c00394_si_001.pdf [file np4c00394_si_001.pdf]

Supporting information

Efficient and selective biosynthesis of a precursor-directed FK506 analogue; paving the way for click chemistry

Dušan Goranovič,[#] Branko Jenko,[#] Barbara Ramšak,[&] Ajda Podgoršek Berke,[#] Leon Bedrač,[#] Jaka Horvat,[#] Martin Šala,[¥] Damjan Makuc,[¥] Guilhermina M. Carriche,[Ł] Luana Silva,[Ł] Aleksandra Lopez Krol,[Ł] Alen Pšeničnik,[&] María Beatriz Durán Alonso,[Ω] Martina Avbelj,[&] Stojan Stavber,[Σ] Janez Plavec,[¥,⊥,□] Tim Sparwasser,[Ł] Rolf Müller,[Ƶ] Gregor Kosec,[#,⊂] Štefan Fujs,[#,⊂] Hrvoje Petković[&,*]

[#]Acies Bio, d.o.o., 1000 Ljubljana, Slovenia

[&]University of Ljubljana, Biotechnical Faculty, Department of Food Science and Technology, 1000 Ljubljana, Slovenia

[¥]National Institute of Chemistry, 1000 Ljubljana, Slovenia

[Ł]Institute of Medical Microbiology and Hygiene and Research Center for Immunotherapy (FZI), University Medical Center of the Johannes Gutenberg-University, Mainz 55131, Germany; Institute of Infection Immunology, TWINCORE,

Centre for Experimental and Clinical Infection Research; a Joint Venture Between the Medical School Hannover (MHH) and the Helmholtz Centre for Infection Research (HZI), Hannover 30625, Germany.

[Ω] Department of Biochemistry and Molecular Biology and Physiology, University of Valladolid, Valladolid, Spain; 47003 Valladolid, Spain.

[Σ] Department of Physical and Organic Chemistry, Jožef Stefan Institute, 1000 Ljubljana, Slovenia.

[⊥] EN → FIST Centre of Excellence, Trg Osvobodilne fronte 13, SI-1000 Ljubljana, Slovenia.

[□] Faculty of Chemistry and Chemical Technology, University of Ljubljana, Večna pot 113, SI-1000 Ljubljana, Slovenia.

[Ƶ] Helmholtz Institute for Pharmaceutical Research Saarland (HIPS), Helmholtz Centre for Infection Research (HZI), and Department of Pharmacy, Saarland University, 66123, Saarbrücken, Germany.

[⊂] Centre of excellence for Integrated Approaches in Chemistry and Biology of Proteins (CIPKeBiP), Jamova 39, SI-1000 Ljubljana, Slovenia.

[*] Corresponding authors: Hrvoje Petković, hrvoje.petkovic@bf.uni-lj.si

1. Synthesis of propargylmalonyl-SNAC (S,S-bis(2-acetamidoethyl)2-(prop-2-yn-1-yl)propanebis(thioate)

The synthesis of propargylmalonyl-SNAC was performed in three steps, as presented in Figure 3. A detailed procedure for its synthesis is provided below:

1.1 Step 1: Synthesis of 2-(prop-2-yn-1-yl)malonic acid

5.315 g (31.23 mmol, 5.0 mL) dimethyl 2-(prop-2-yn-1-yl)malonate was added to a solution of 3.192 g NaOH in 5.0 mL demineralized water cooled in an ice bath and the solution was stirred at room temperature overnight; next, 15 mL of demineralized water was added to dissolve the yellow precipitate that had formed. The solution was acidified using 28 mL of 20% aqueous HCl, 30 mL of demineralized water was added and the solution was extracted by diethyl ether (3 x 100 mL). The joined organic phases were dried over anhydrous Na_2SO_4, the solvent was removed under reduced pressure and 4.358 g (98%) of white crystalline material was obtained.

^{1}H NMR (298 MHz, Chloroform-*d*) δ 11.56 (s, 2H), 3.55 (t, *J* = 7.4 Hz, 1H), 2.79 (dd, *J* = 7.4, 2.7 Hz, 2H), 2.03 (t, *J* = 2.7 Hz, 1H).

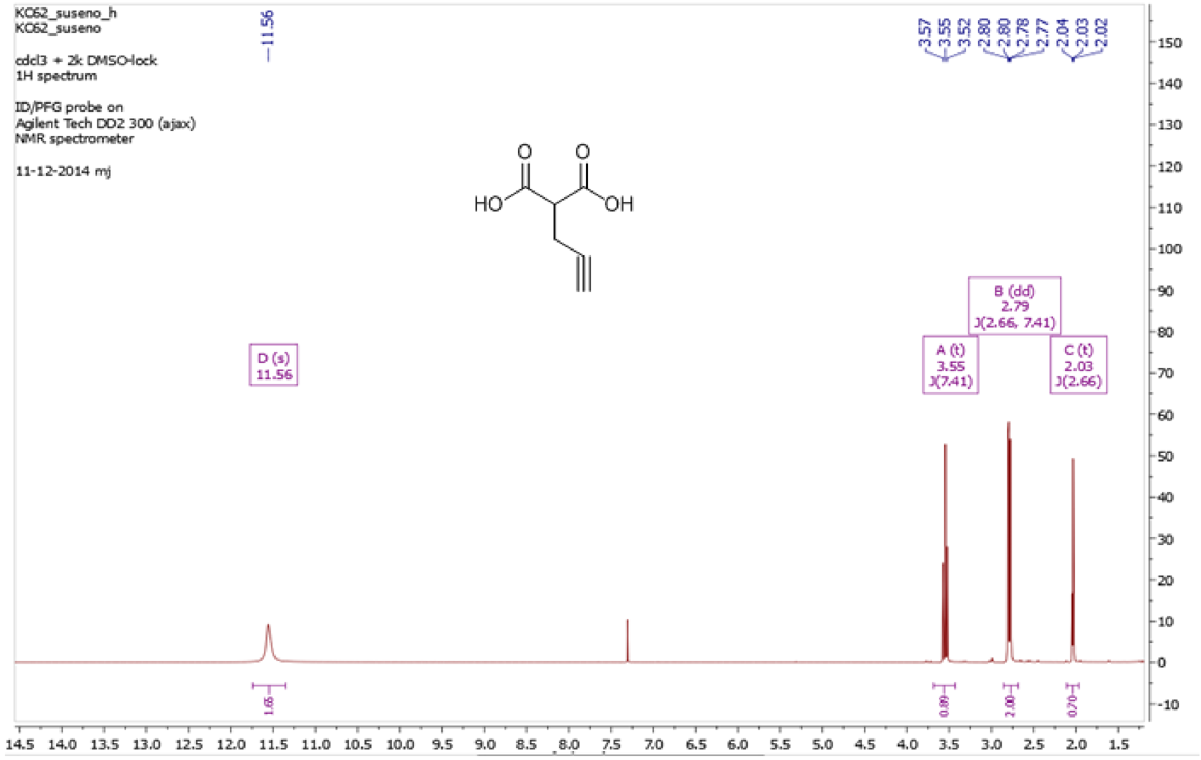

Figure S1: ^{1}H NMR spectra of **2-(prop-2-yn-1-yl)malonic acid.**

1.2 Step 2: Synthesis of 2-(prop-2-yn-1-yl)malonyl chloride

4.0 g alylmalonic acid (28.15 mmol) was introduced into a 250 mL round bottom glass vessel ventilated by dry argon and suspended in 47 mL of dry dichlomethane; 300 mL of dry dimethylformamide was injected into the suspension and activated molecular sieves were added. The reaction mixture was cooled on an ice bath and 12.07 g (95 mmol, 8.15 mL) of oxalyl chloride was added in drops while stirring for a period of 15 minutes. The mixture was stirred at room temperature overnight and filtered; crude 2-(prop-2-yn-1-yl)malonyl chloride (brown liquid, 4.662 g, 92%) was isolated once the volatile solvents had

evaporated under reduced pressure and used in the following procedures without any purification.
^{1}H NMR (298 MHz, Chloroform-*d*) δ 4.39 (t, *J* = 7.3 Hz, 1H), 2.98 (dd, *J* = 7.2, 2.6 Hz, 2H), 2.20 (t, *J* = 2.6 Hz, 1H).

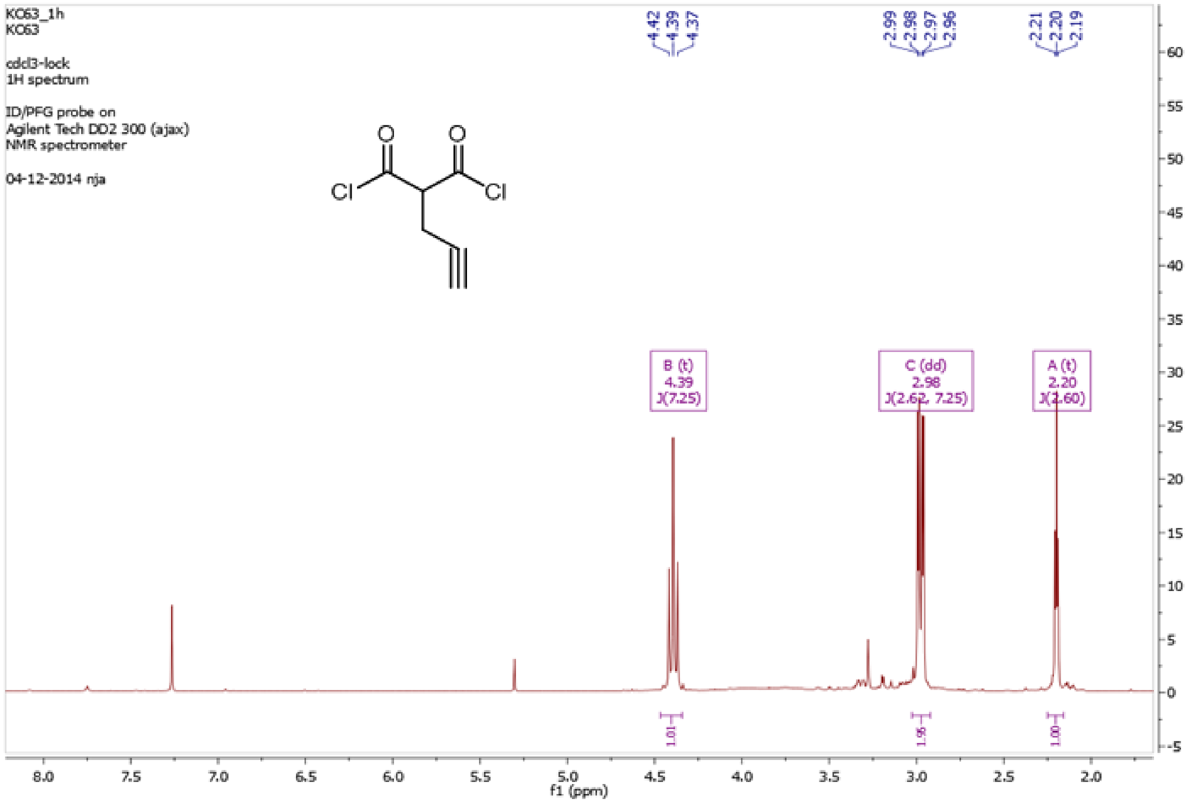

Figure S2: ^{1}H NMR spectra of **2-(prop-2-yn-1-yl)malonyl chloride.**

1.3 Step 3: *Synthesis of S,S*-bis(2-acetamidoethyl) 2-(prop-2-yn-1-yl)propanebis(thioate)

4.642 g (26,04 mmol) of 2-(prop-2-yn-1-yl)malonyl chloride was transferred to a three-necked round bottom glass vessel, 120.5 ml of dry THF was added and the solution was cooled in an ice bath. 6.2 g (52.08 mmol, 5.54 mL) of N-acetylcisteamine was dropwise added while stirring at 0° C during a period of 30 minutes and then an additional 10.13 mL (72.63 mmol) of trimethylamine was added within a period of 10 minutes. The reaction mixture was stirred in an ice bath for an additional 3 hours, after which the solvents were evaporated. 435 mL of demineralized water was added to the residue an the extraction was carried out using dichloromethane (3 x 360 mL). The organic phases were combined and dried over anhydrous Na_2SO_4. The solvent was removed under reduced pressure and the crude material was purified by column chromatography (SiO_2, $CHCl_3$ / MeOH = 92:8); 4.28 g (48%) to yield a brown oily.

^{1}H NMR (303 MHz, Chloroform-*d*) δ 6.77 (s, 2H), 4.04 (t, *J* = 7.5 Hz, 1H), 3.47 (td, *J* = 9.9, 8.8, 3.7 Hz, 4H), 3.19 (dt, *J* = 13.8, 5.8 Hz, 2H), 3.12 – 2.99 (m, 2H), 2.81 (dd, *J* = 7.5, 2.6 Hz, 2H), 2.09 (t, *J* = 2.6 Hz, 1H), 1.98 (s, 6H).
^{13}C NMR (76 MHz, cdcl3) δ 192.16, 170.70, 79.01, 71.17, 66.12, 38.70, 29.53, 22.96, 18.96.
MS (ESI) m/z 345.1 ((M+H)$^{+}$, 100 %).
MS HRMS (ESI) m/z $C_{14}H_{21}N_2O_4S_2$ calculated 345.0943 (MH^{+}), measured 345.0941 (MH^{+}).

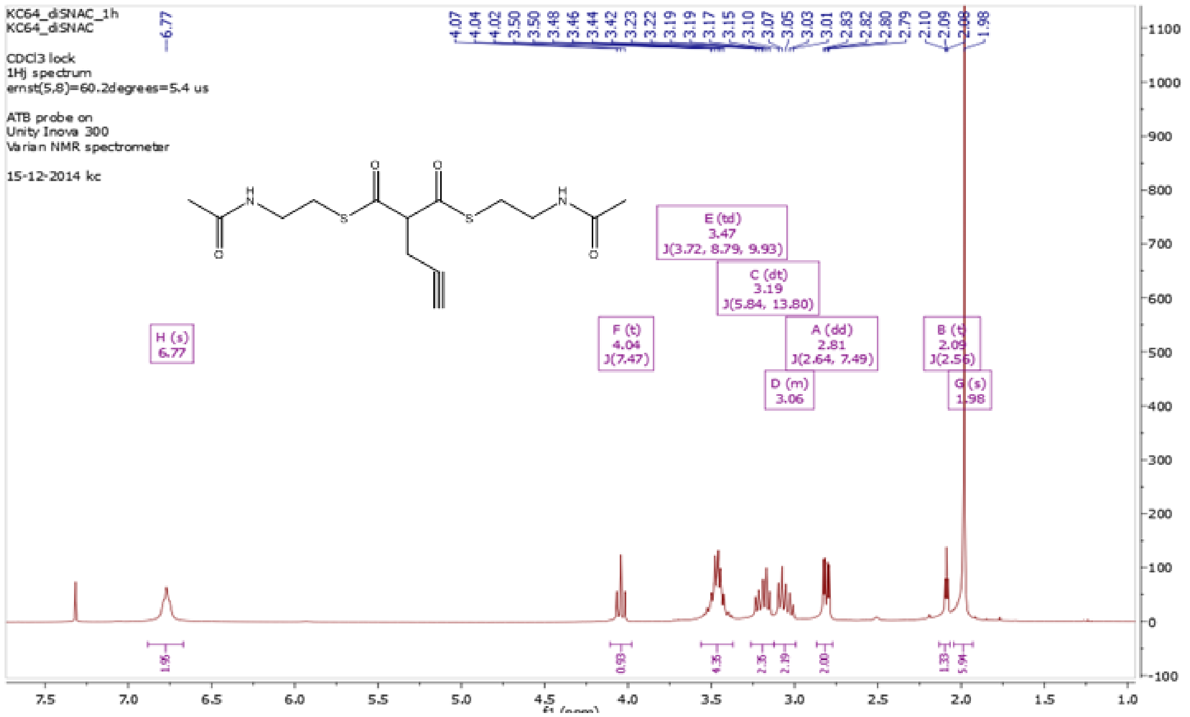

Figure S3: ^{1}H NMR spectra of ***S*,*S*-bis(2-acetamidoethyl) 2-(prop-2-yn-1-yl)propanebis(thioate).**

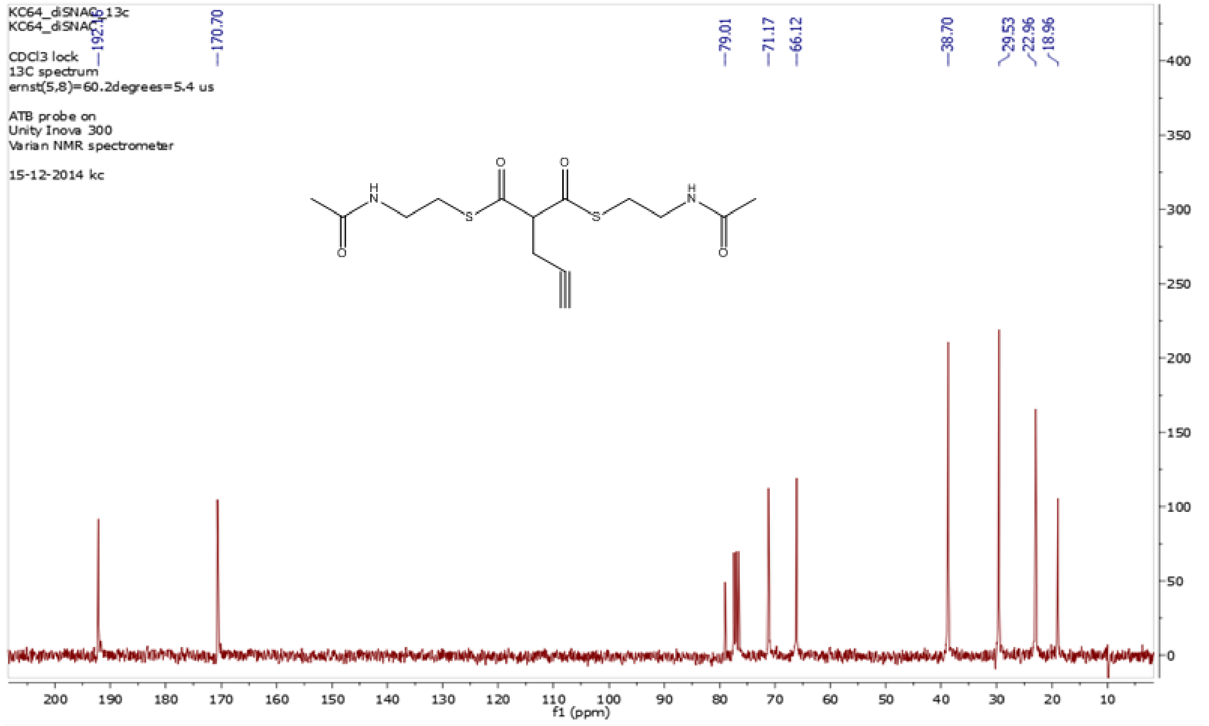

Figiure S4: ^{13}C NMR spectra of ***S*,*S*-bis(2-acetamidoethyl) 2-(prop-2-yn-1-yl)propanebis(thioate).**

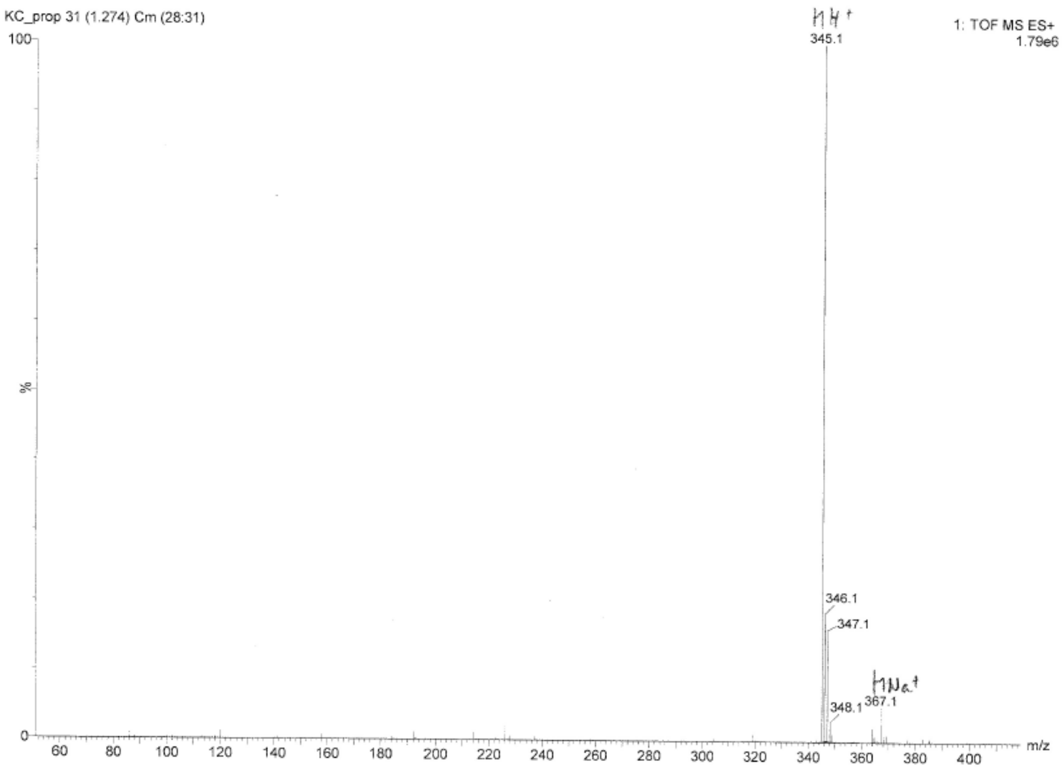

Figure S5: MS spectra of ***S,S*-bis(2-acetamidoethyl) 2-(prop-2-yn-1-yl)propanebis(thioate).**

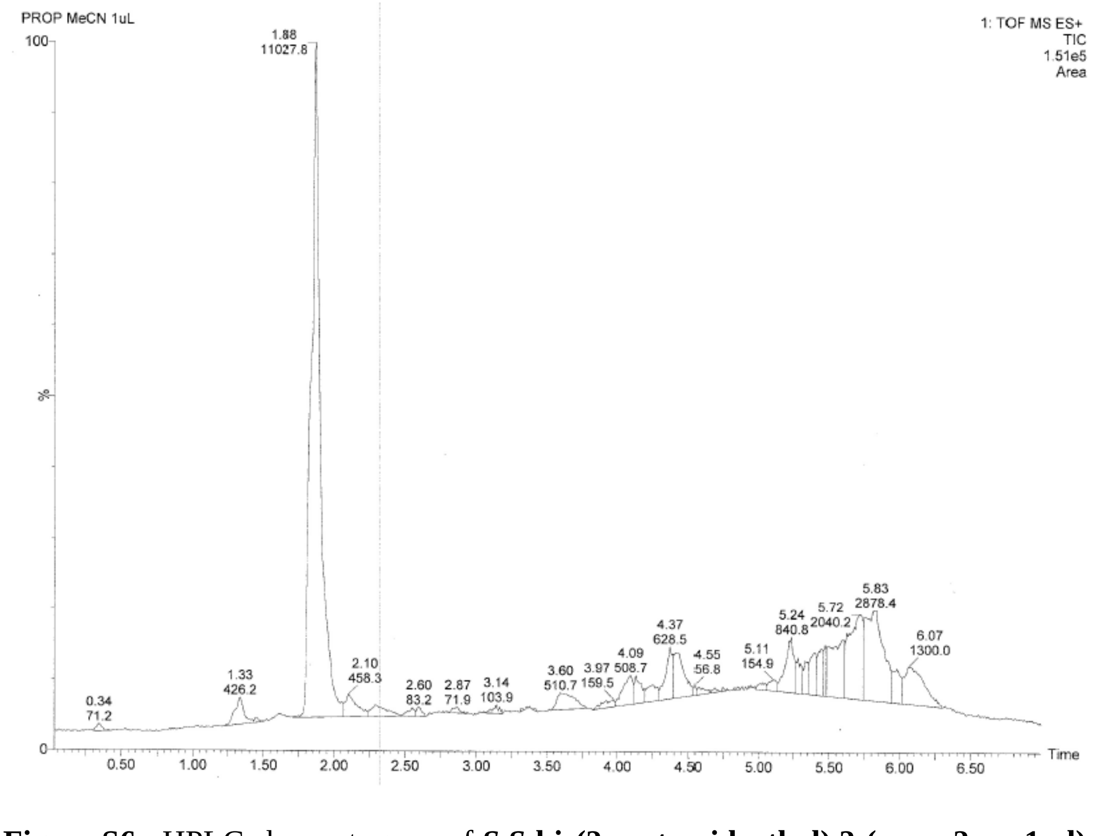

Figure S6: HPLC chromatogram of ***S,S*-bis(2-acetamidoethyl) 2-(prop-2-yn-1-yl)propanebis(thioate).**

2. HPLC and LC/MS analyses of crude material and of the purified propargyl FK506 analogue

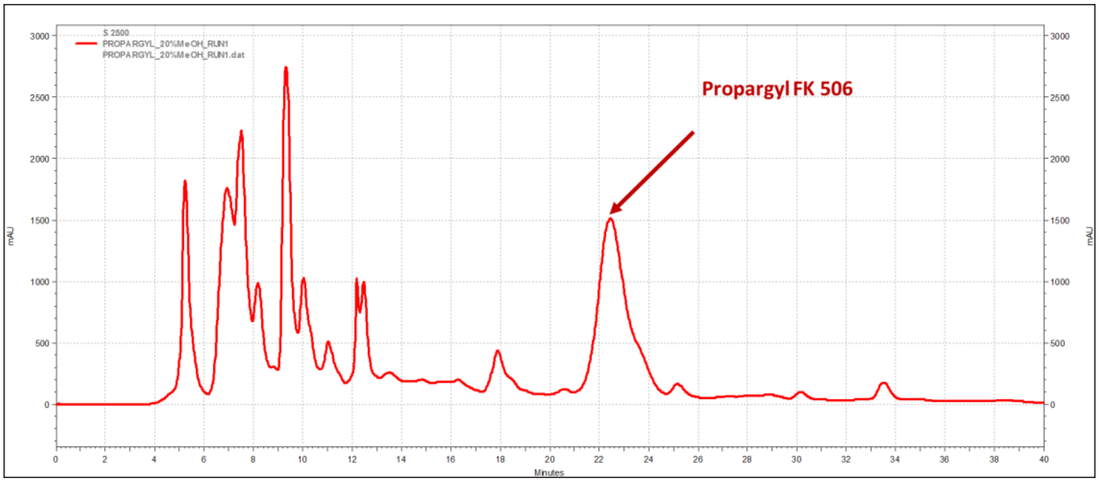

Figure S7: Typical UV chromatogram of crude material purified by preparative HPLC.

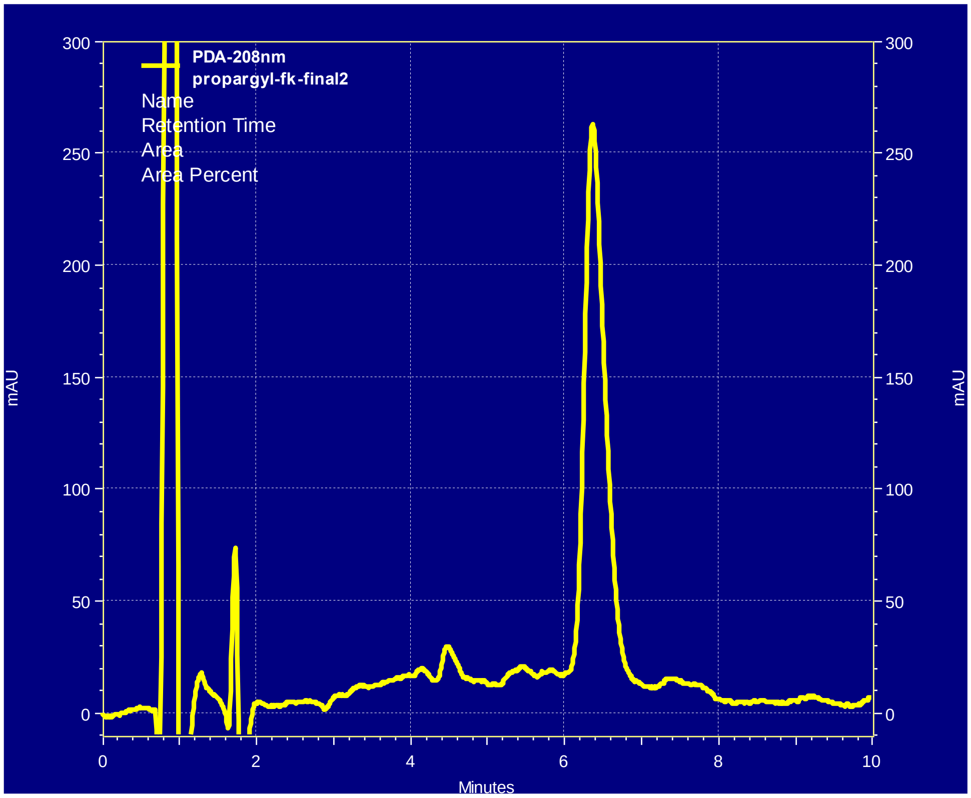

Figure S8: HPLC chromatogram of isolated C21 propargyl FK506 (6,2 min).

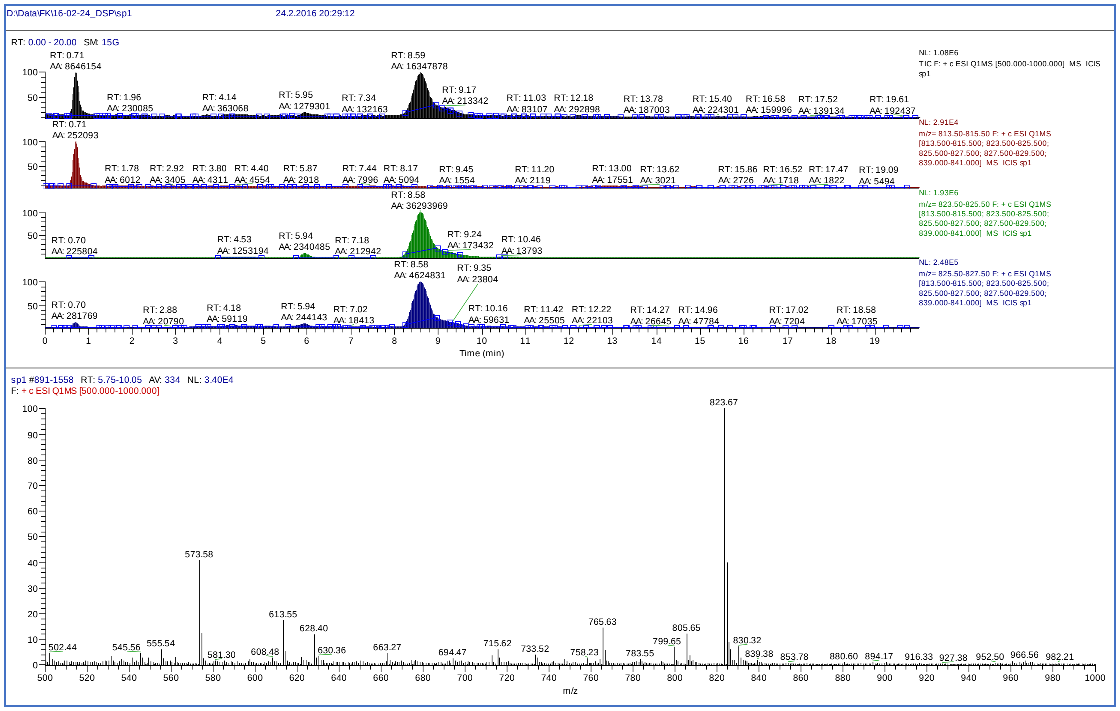

Figure S9: LC-MS analysis of the final product isolated by preparative HPLC.

3. Optimization of the chemobiosynthetic procedure by applying different regimes of allylmalonyl-SNAC feeding

Table S1. The effect of different feeding regimes (feeding concentrations) on the packed cell volume (PCV) and pH value of the culture during the bioprocess.

	S. tsukubaensis NRRL 1848		*S. tsukubaensis ΔallR*	
	Feeding allylmalonyl-SNAC			
Addition of allylmalonyl-SNAC [g/L/day]	pmV	pH	pmV	pH
0	40	7,3	42	7,3
0,5	36	7,0	30	7,4
1,0	20	6,0	20	6,0
1,5	14	6,1	16	6,0

4. *NMR analysis of* the propargyl-FK506 analogue.

Series of 1D and 2D NMR spectra of **propargyl-FK506** were acquired on 800 MHz NMR spectrometers, allowing its structural characterization. ^{1}H and ^{13}C NMR chemical shifts of **propargyl-FK506** are reported in Table 2. Its chemical structure is in full agreement with the structure presented in Figure 1. Two sets of signals were observed in ^{1}H and ^{13}C NMR spectra, which were attributed to *cis* and *trans* rotamers along the peptide bond. The ratio between *cis* and *trans* rotamers is 2:1 for **propargyl-FK506**. *Cis* and *trans* rotamers along the amide bond can be distinguished with respect to characteristic

differences in chemical shifts of C2 and C6 (δ_C 57.5 and 39.86 ppm for *cis* rotamer; δ_C 53.36 and 45.01 ppm for *trans* rotamer).

The sample was dissolved in deuterated pyridine (Pyridine-d_5). The following 1D and 2D NMR spectra were acquired for sample **propargyl-FK506** on an 800 MHz NMR spectrometer to determine its chemical structure:

1. ^{1}H NMR spectrum (Figure S10),
2. ^{13}C NMR spectrum (Figure S11),
3. ^{1}H-^{1}H gCOSY spectrum (Figure S12),
4. ^{1}H-^{1}H TOCSY spectrum, mixing time 80 ms (Figure S13),
5. ^{1}H-^{13}C gHSQC spectrum (Figure S14),
6. ^{1}H-^{13}C gHMBC spectrum (Figure S15),
7. Part of the ^{1}H NMR spectrum of propargyl-FK506 (Figure S16) in Pyridine-d_5 at 25 °C, between 3.5 and 6.2 ppm.
8. Part of the ^{13}C NMR spectrum of propargyl-FK506 in Pyridine-d_5 at 25 °C, between 10 and 40 ppm (Figure S17).

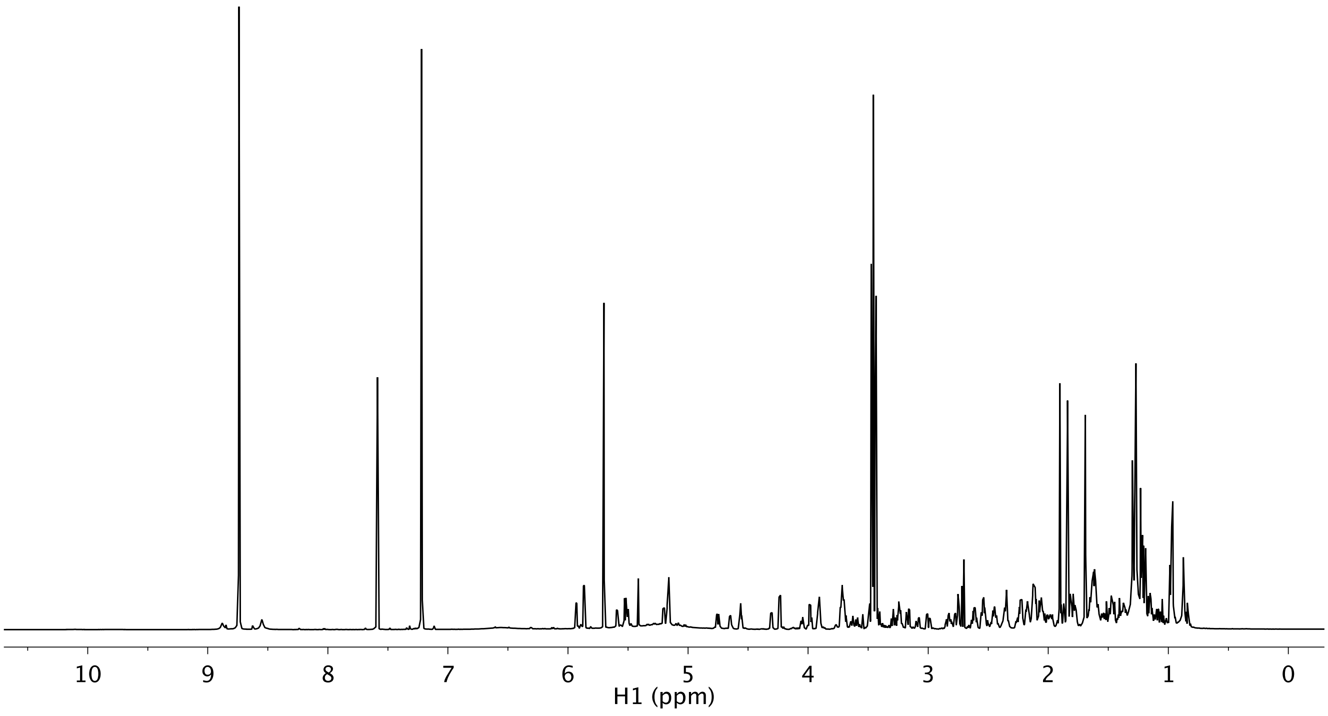

Figure S10. ^{1}H NMR spectrum of **propargyl-FK506** in Pyridine-d_5 at 25 °C.

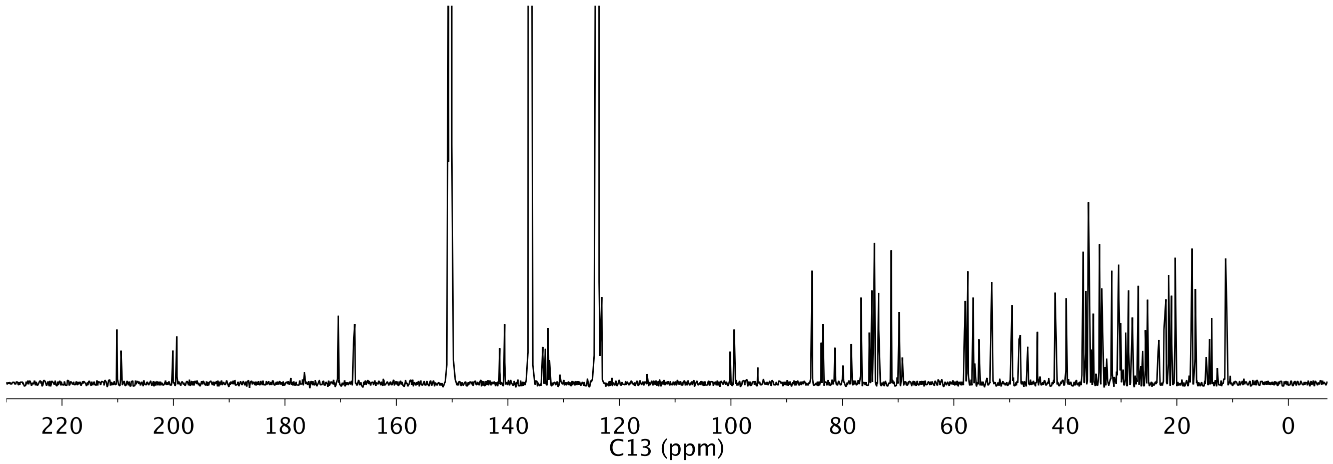

Figure S11. ^{13}C NMR spectrum of **propargyl-FK506** in Pyridine-d_5 at 25 °C.

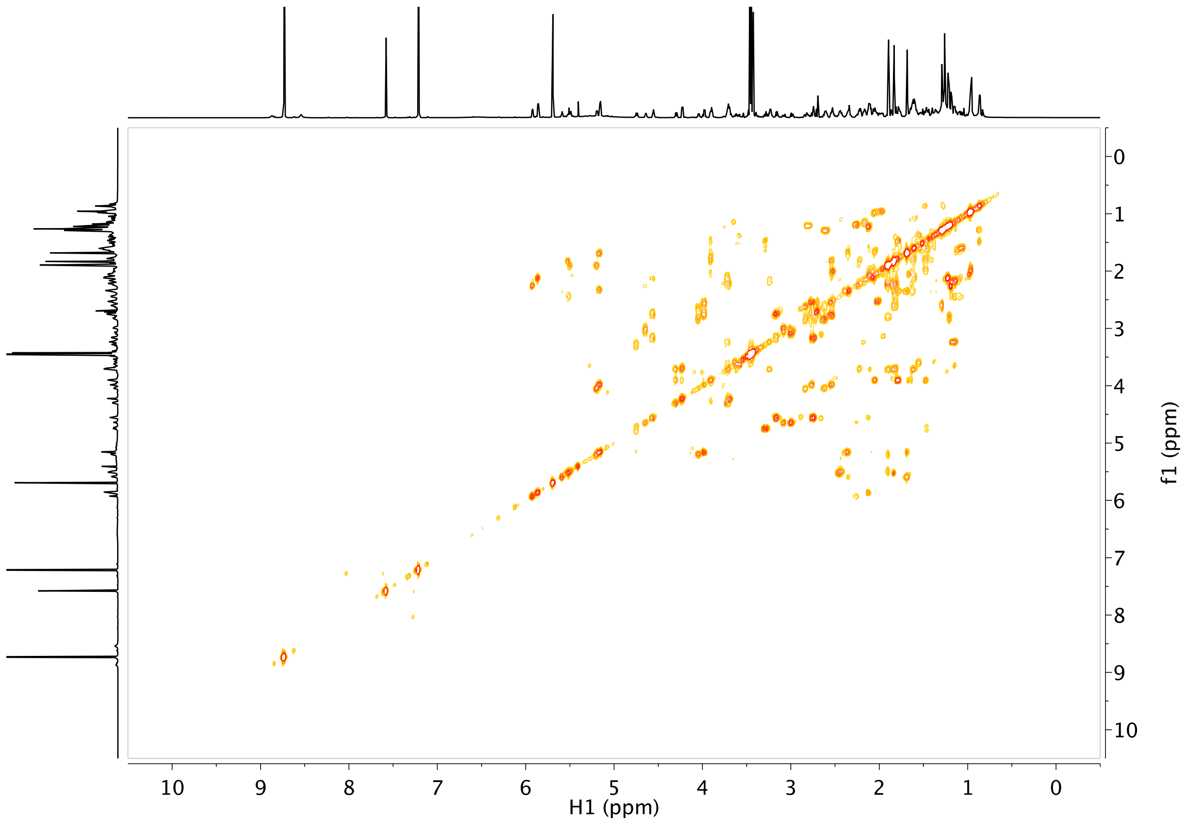

Figure S12. ^{1}H-^{1}H gCOSY NMR spectrum of **propargyl-FK506** in Pyridine-d_5 at 25 °C.

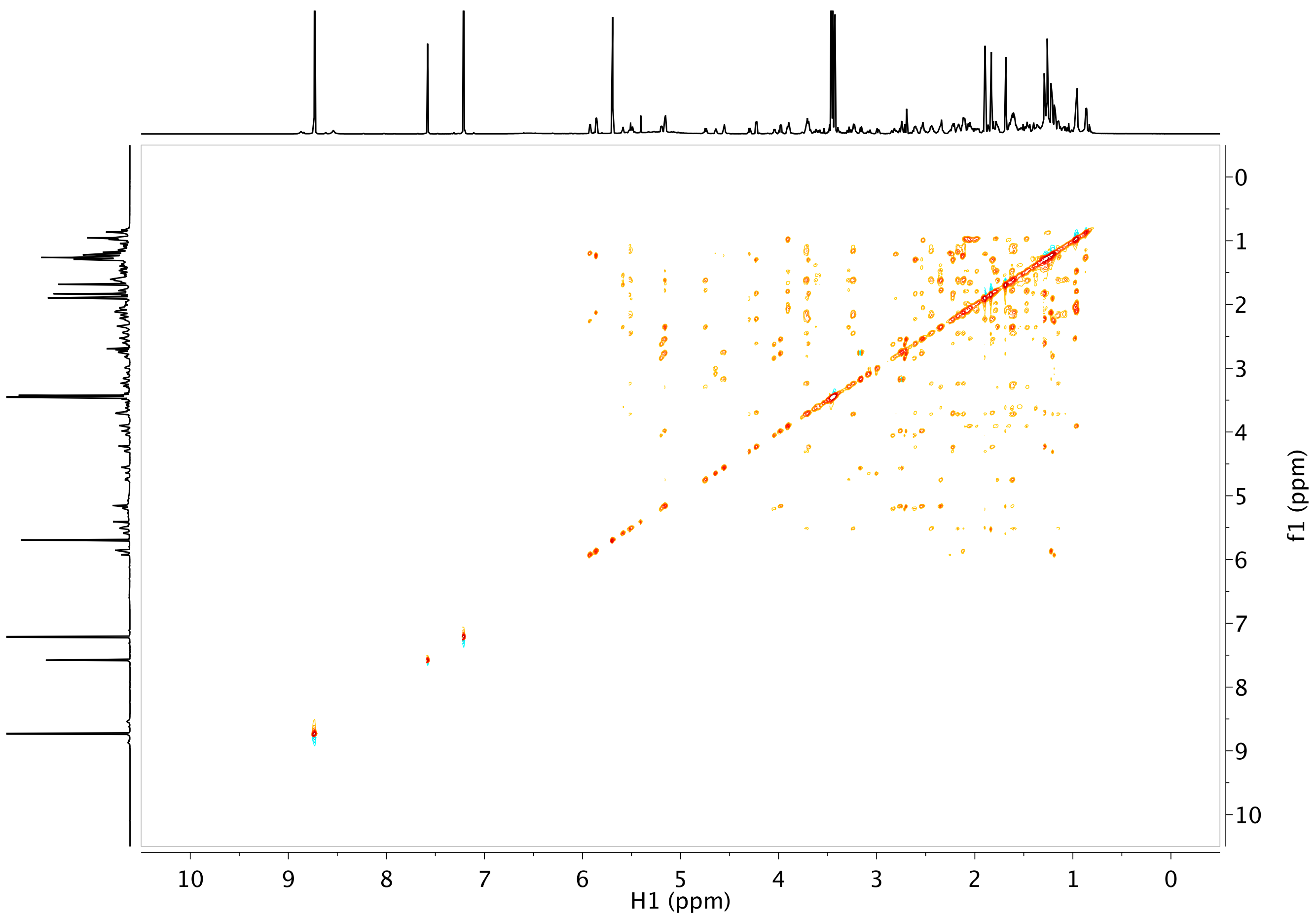

Figure S13. ^{1}H-^{1}H TOCSY spectrum of **propargyl-FK506** in Pyridine-d_5 at 25 °C, mixing time 80 ms.

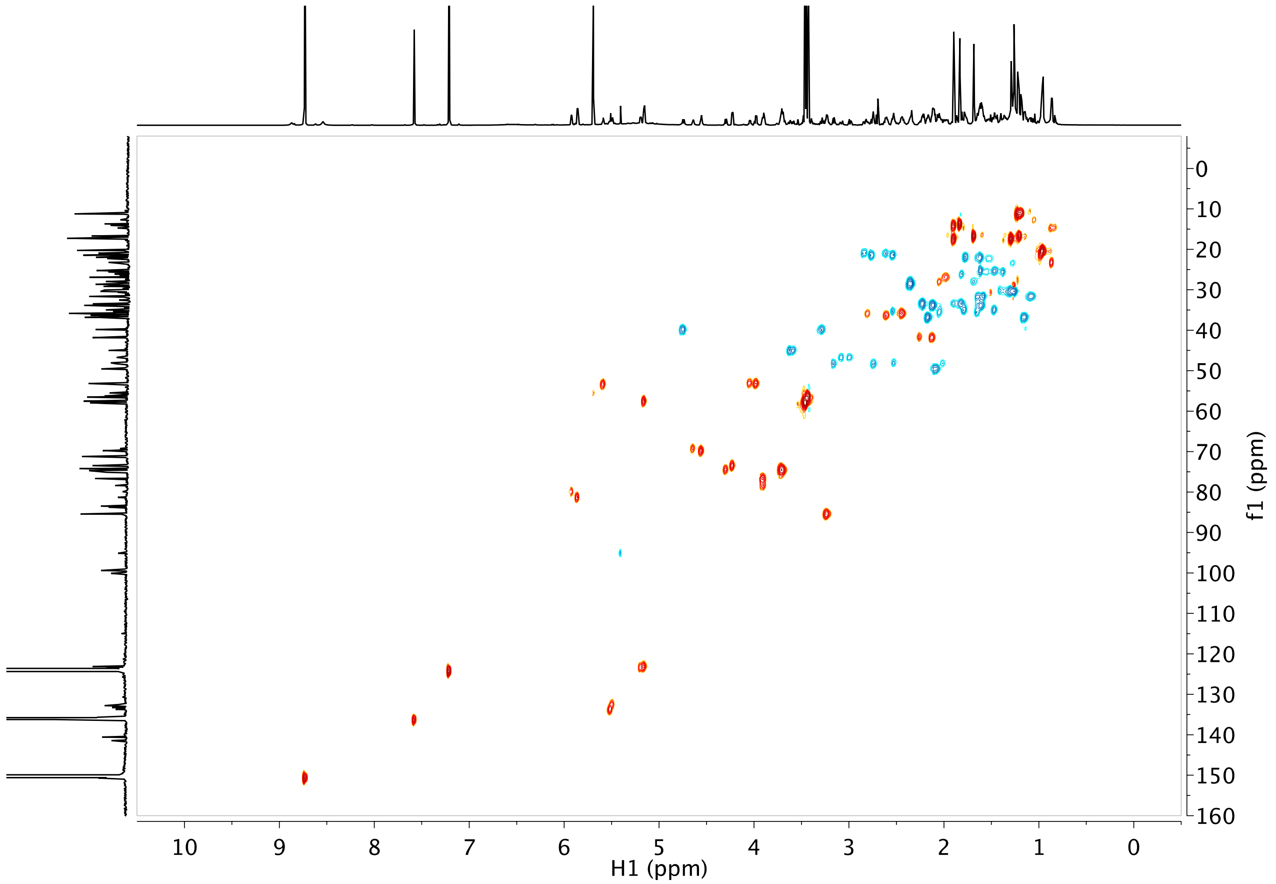

Figure S14. ^{1}H-^{13}C gHSQC NMR spectrum of **propargyl-FK506** in Pyridine-d_5 at 25 °C.

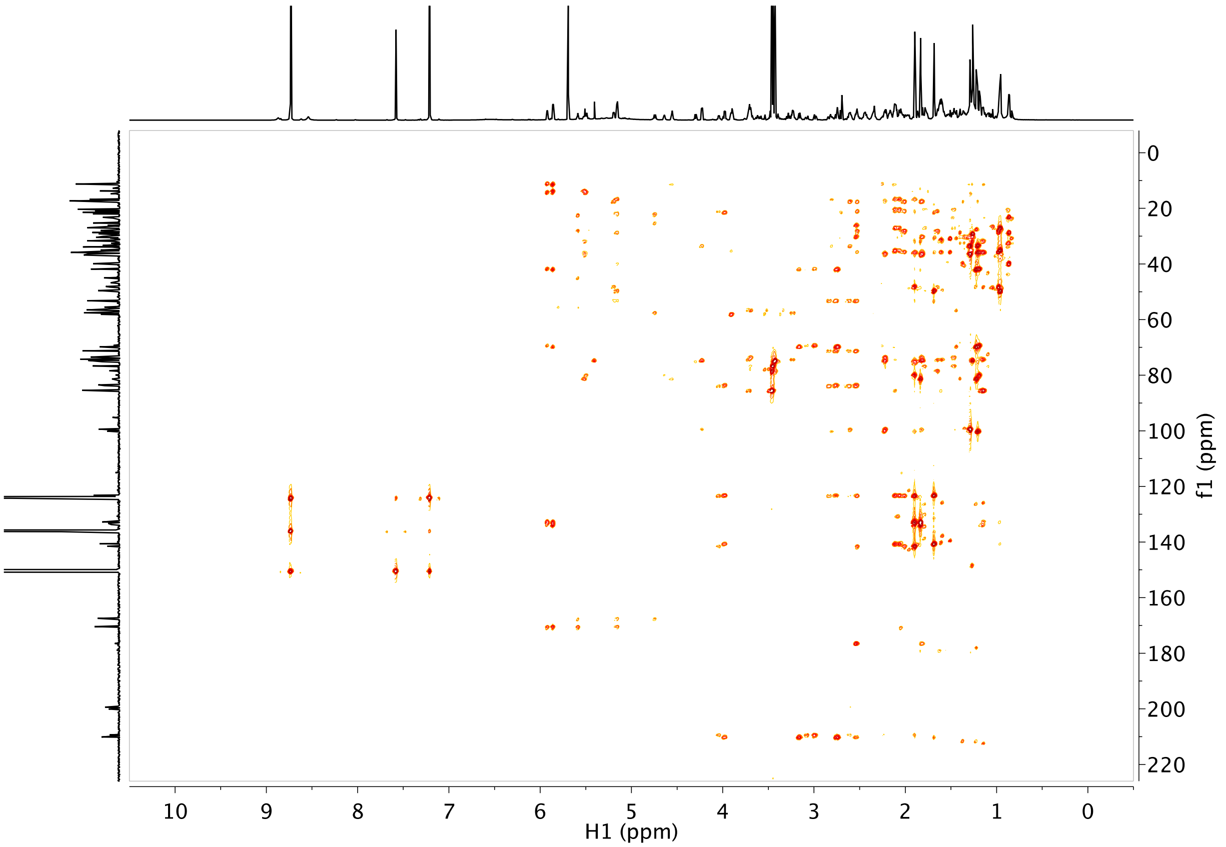

Figure S15. ^{1}H-^{13}C gHMBC NMR spectrum of **propargyl-FK506** in Pyridine-d_5 at 25 °C.

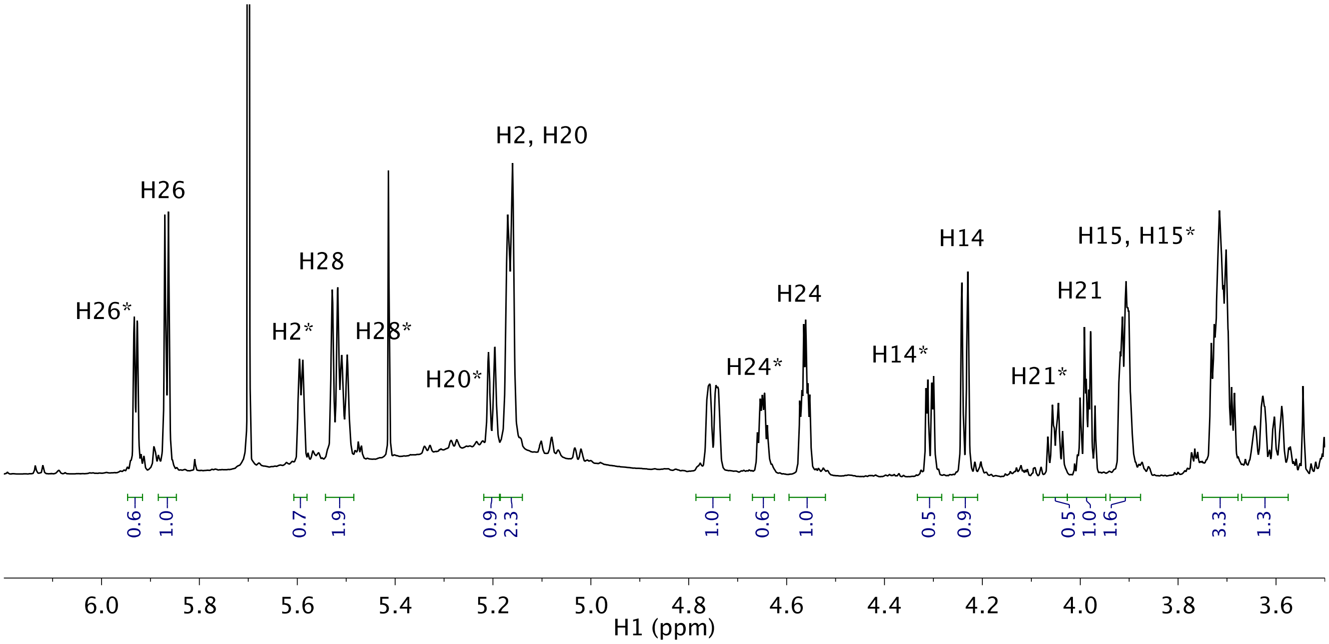

Figure S16. Part of the ^{1}H NMR spectrum of **propargyl-FK506** in Pyridine-d_5 at 25 °C, between 3.5 and 6.2 ppm, showing clearly distinguishable signals from the major and the minor species (the signals from the minor species are denoted with *).

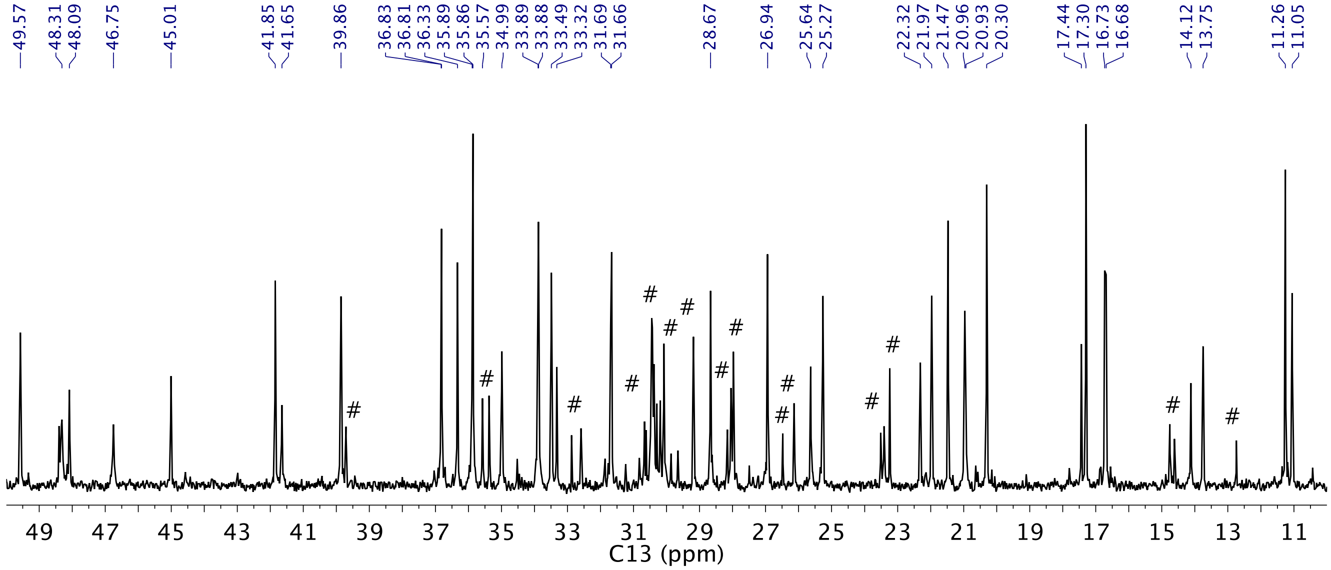

Figure S17. Part of the ^{13}C NMR spectrum of **propargyl-FK506** in Pyridine-d_5 at 25 °C, between 10 and 40 ppm, where signals attributed to impurities are marked with hashtags (#). Peaks noted with chemical shifts were attributed to major or minor species of the tacrolimus analogue.

5. Evaluation of the biological activity of C21 propargyl-FK506

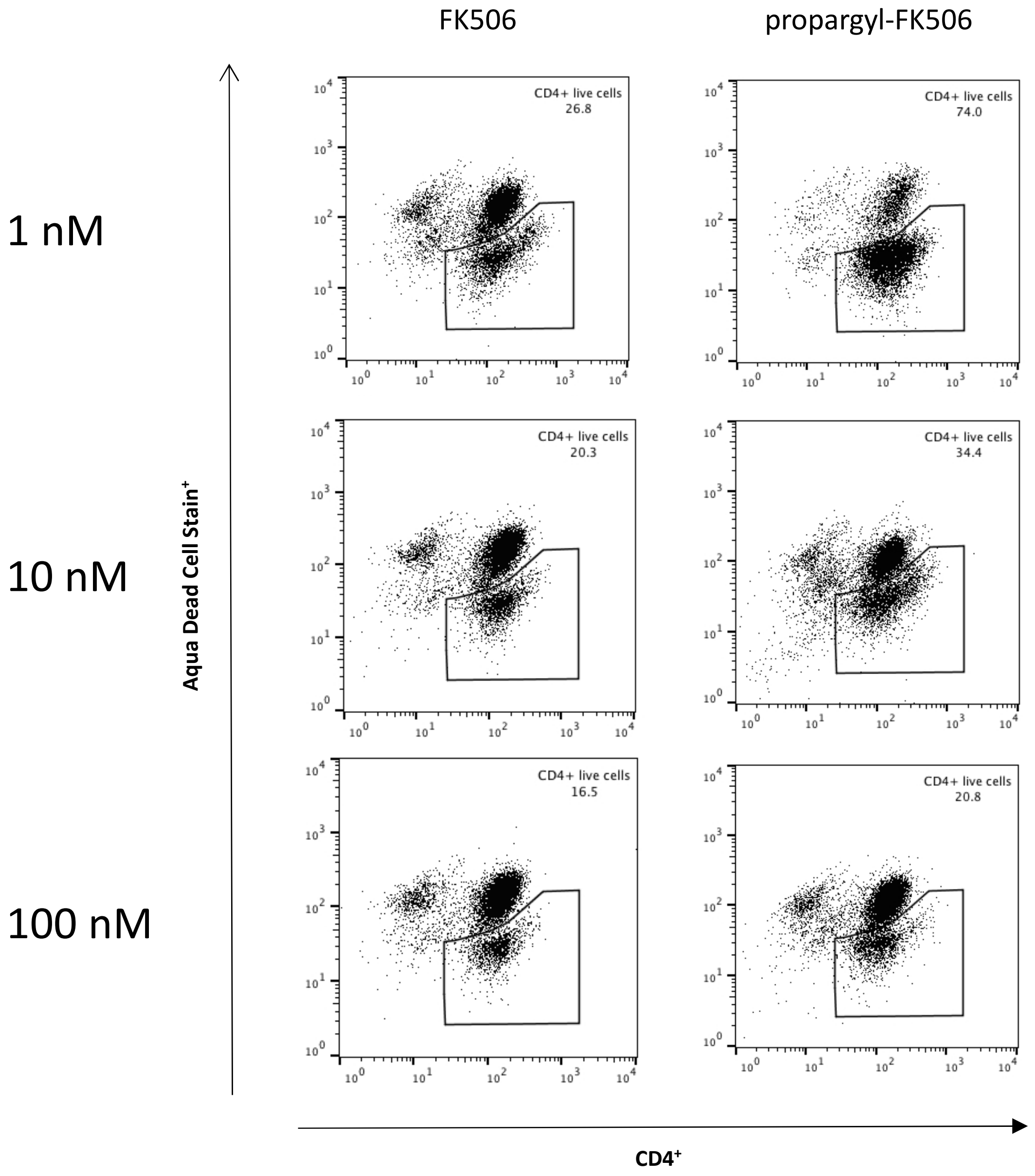

Figure S18: Inhibition of T cell proliferation (toxicity) by FK506 and C21 propargyl FK506. Magnetic bead-enriched $CD4^+$ T cells were cultured for 4 days in the presence or absence of FK506 and C21 propargyl-FK506, added at varying concentrations: 0.5nM, 1nM, 5nM, 10nM and 100nM. After 4 days of culture, $CD4^+$ live cells were analysed by flow cytometry.

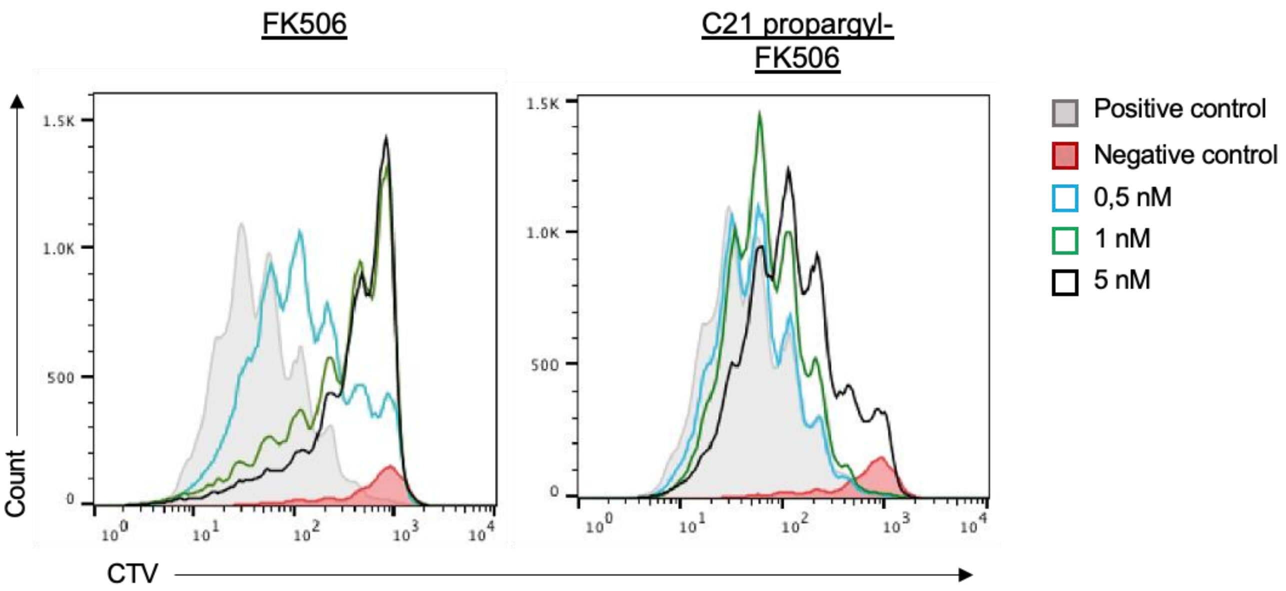

Figure S19. Proliferation was assessed by flow cytometry using the CellTrace™ Violet Proliferation kit, following 4 days of co-culture.

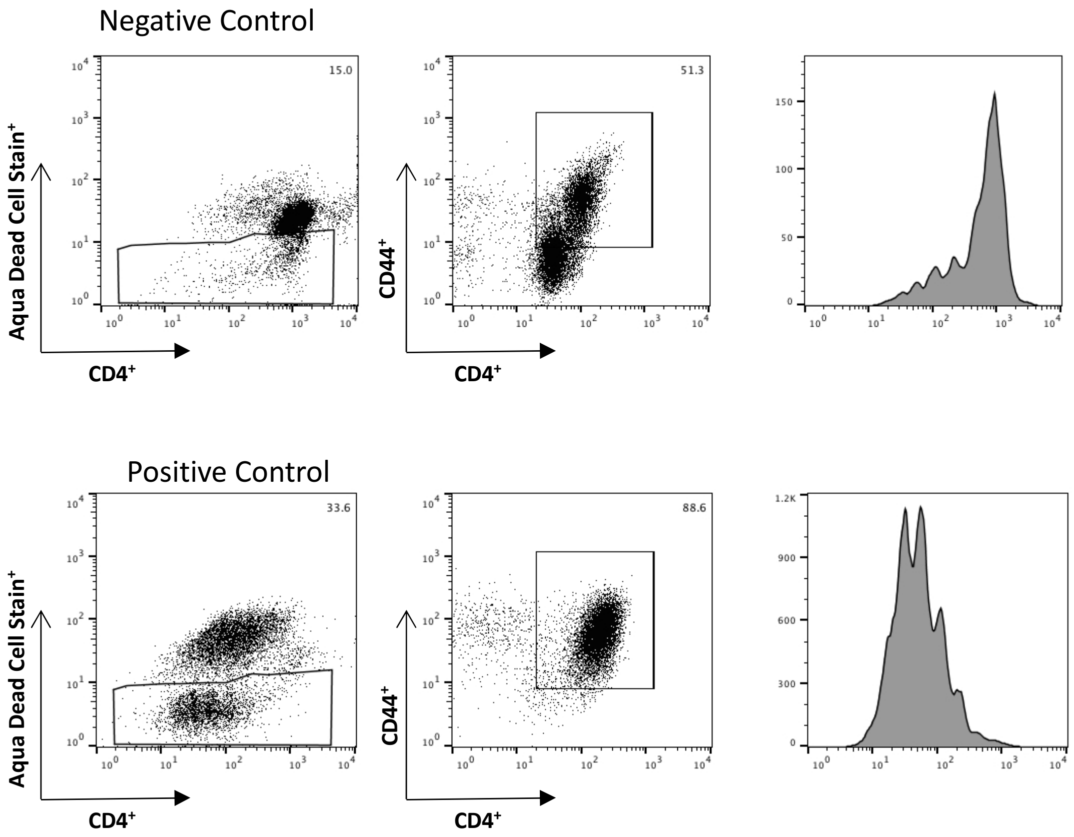

Figure S20: Gating strategies are described.

6. Additional structural confirmation of C21 propargyl-FK506 by LC-MS/MS analysis

After cultivation was completed, the broth was extracted with the equal volume of methanol (1:1). To confirm the presence of the C21 propargyl-FK506 analogue, in the fed cultivation broths, we carried out LC-MS/MS analysis. We used the Agilent 1100 series LC-MS system coupled with Waters Micromass Quattro micro detector using reversed phase column (Gemini CI8 column, 5 μη , 150 mm 2 mm i.d.) from Phenomenex. The separation was performed at a flow rate of 0.250 ml/min by gradient elution with 0.5 % TFA as solvent A and acetonitrile as solvent B. The gradient program was: 60 % A, 0 min; 60-20 % A, 0-17 min; 20-60 % A, 17-18 min; 60 % A, 18-30 min and the injection volume 10 μΐ at temperature of the column 45 °C was used.
The mass selective detector (Waters, Quattro micro API) was equipped with an electrospray ionisation using a cone voltage of 20 V and capillary voltage of 3.5 kV for positive ionization of the analytes. Dry nitrogen was heated to 350 °C, the drying gas flow was 400 1/h and collision energy was 20 eV. When the presence of the original FK506 compound is detected using an ESI+ positive mode, an ion of *m/z* = 826.5 that corresponds to a capture of a sodium ion ($[M+Na]^+$ is most intensive in accordance with the results of other investigators.

For FK506 quantification, selected reaction-monitoring mode can be used and the transition FK506 m/z 826.5$[M+Na]^+$ m/z 616.4 can be observed. In order to determine the presence of C21 propargyl-FK506 we used a similar approach, however, the molecular masses of both ions in multiple reaction-monitoring transition were adapted according to the predicted molecular mass of each analogue (Table S2).

Table S2: LC-MS/MS data confirming the presence of novel FK506 analogue in fed fermentation broths.

Extender unit fed	Extender unit name	Observed m/z $[M+Na]^+$	Observed m/z [transition ion]$^+$	Molecular mass
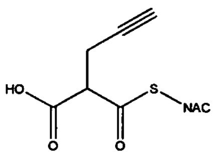	Propargylmalonyl-SNAC	824.5	614.4	802

For the compounds FK506, FK520, and the C21 propargyl-FK506 analogue, MS2 experiments were conducted using HPLC-MS. The $[M+Na]^+$ ions were selected for fragmentation due to their significantly higher abundance compared to their protonated counterparts. Additionally, the fragmentation patterns of these sodiated ions are more distinct and informative, as demonstrated in the manuscript by Mevizou et al. (Pharmacological Research, Volume 209, November 2024, 107438 https://doi.org/10.1016/j.phrs.2024.107438) (**33**).
The observed transitions were *m/z* 826.5 → 616.2, 814.5 → 604.2, and 824.5 → 614.2. These fragments are consistent with the structural modifications on the original FK506 scaffold, according to fragmentation of FK506 described by Mevizou et al. Therefore, based on the fragmentation data, we can confidently conclude that the compound is indeed the C21 propargyl-FK506 analogue. This supports the structural integrity and modifications of the compounds under investigation.
